# Study on the disease burden of lip and oral cancer attributable to tobacco use: based on the 2021 Global Burden of Disease study

**DOI:** 10.3389/fonc.2025.1690271

**Published:** 2025-11-07

**Authors:** Shu’ang Shu, Shuping Weng, Jinchao Gu

**Affiliations:** Department of Stomatology, The Affiliated Yangming Hospital of Ningbo University, Yuyao, China

**Keywords:** tobacco, lip and oral cavity cancer, cross-national inequality, GBD, epidemiological trends

## Abstract

**Background:**

To analyze the global, regional, and national epidemiological trends of lip and oral cancer (LOC) diseases attributable to tobacco from 1990 to 2021, with an emphasis on health inequalities.

**Methods:**

Utilizing the Global Burden of Disease (GBD) 2021 study, we evaluated the temporal trends in tobacco-attributable LOC mortality and disability-adjusted life years (DALYs). We further analyzed how LOC mortality and DALYs varied by age, period, and birth cohort. The Bayesian Age-Period-Cohort (BAPC) model was employed to project trends from 2021 to 2050. Additionally, decomposition analysis was conducted to identify key drivers of change, and health inequalities were assessed. Finally, frontier analysis was performed across global countries and regions.

**Results:**

From 1990 to 2021, tobacco-attributable LOC mortality and DALYs exhibited a significant declining trend globally (estimated annual percent change of -0.49 and -0.61, respectively). Population growth was the primary driver of increasing burden, while epidemiological transition mitigated the burden. Projections indicate that the burden will continue to decline by 2050, with low socio-demographic index (SDI) regions experiencing significantly higher burden compared to high SDI regions. Age effects showed a stepwise increase in mortality with advancing age, period analysis confirmed the sustained reduction in tobacco-related harm, and cohort studies revealed significantly lower risk among later-born populations. Cross-national analysis revealed a narrowing gap in burden between high and low SDI countries, yet countries such as Pakistan and Palau still exhibit substantial health benefit gaps.

**Conclusion:**

Although the global burden of tobacco-attributable LOC diseases shows a slow declining trend, low and middle SDI regions continue to maintain high burden levels. Strengthening tobacco control strategies in these regions are essential to narrow health disparities.

## 1 Introduction

Lip and oral cancer (LOC) is one of the most common malignant tumors in the head and neck region, typically occurring in the lips, tongue, gums, floor of the mouth, and palate (1, 2). According to the latest statistics, an estimated 377,713 new cases of LOC were diagnosed globally in 2020, making it the sixth most common cancer worldwide (3, 4). Despite significant improvements in cancer diagnosis and treatment for most oral cancer patients over the past few decades, the 5-year survival rate remains below 50% (5). A study based on the Indian population revealed that the 5-year survival rate for oral cancer patients was as low as 32.8% (6). Undetected or inadequately treated early-stage LOC may carry the risk of systemic lymph node metastasis, posing a threat to patients’ health and lives (7, 8). Considering this, a comprehensive assessment of the burden of LOC is crucial for effectively addressing the significant challenges posed by cancer.

The development and progression of LOC cancer are influenced by multiple factors, including tobacco smoking (9), alcohol consumption (10), ultraviolet radiation (11), areca nut chewing (12), and human papillomavirus (HPV) infection (13). Among these, tobacco use is the most significant lifestyle-related risk factor. Tobacco contains numerous carcinogens, such as polycyclic aromatic hydrocarbons, nitrosamines, and comatic amines. Through prolonged exposure, these substances can induce genetic mutations, abnormal proliferation, and impaired apoptosis in oral mucosal epithelial cells, thereby leading to carcinogenesis (14, 15). Studies have shown that smoking increases the incidence of oral cancer by sixfold, whereas smoking cessation significantly reduces oral cancer mortality at both 3 and 5 years (16). The Global Burden of Disease (GBD) study in 2019 indicated that 42.3% of global LOC deaths were attributable to tobacco use (17). Furthermore, in countries such as China, India, Brazil, and Russia, high rates of tobacco consumption have contributed to a substantial increase in LOC cases (18). Therefore, understanding the latest trends and burden of tobacco-related LOC is important for reducing the overall burden of LOC.

The GBD study provides a comprehensive data resource for assessing the health impact of various diseases and risk factors at global, regional, and national levels. However, research specifically examining the disease burden attributable to tobacco and its association with LOC, particularly in-depth analyses based on the most recent GBD data, remains insufficient. Therefore, this study aims to systematically evaluate the disease burden of LOC attributable to tobacco between 1990 and 2021, based on the GBD 2021 database. It further seeks to explore variations in this burden across different regions, countries, sexes, age groups, and socio-demographic index (SDI) levels. The objective is to provide a scientific basis for developing effective prevention and intervention strategies.

## 2 Methods

### 2.1 Data source

The data used in this study were obtained from the GBD 2021 public dataset, which is accessible via the Global Health Data Exchange GBD Results Tool (https://ghdx.healthdata.org/gbd-2021). This database provides comprehensive data for 204 countries and territories, encompassing 371 diseases and injuries, and 88 risk factors. In GBD 2021, LOC is defined according to the International Classification of Diseases, 10th Revision (ICD-10): C00-C08.9 (19). For this study, we extracted mortality rates and DALYs for LOC attributable to tobacco. Age-standardized mortality rates (ASMR) and age-standardized DALY rates (ASDR) were calculated using the GBD 2021 global standard population. The 95% uncertainty intervals (UI) were obtained by resampling the data 1000 times, with the upper and lower bounds determined by the 2.5th and 97.5th percentiles of the uncertainty distribution, respectively. For the GBD study, institutional review board review and approval for waiver of informed consent were granted by the University of Washington (https://www.healthdata.org/research-analysis/gbd).

The SDI is used to assess the level of socio-demographic development, providing a composite measure that incorporates fertility rates in females under 25 years of age, mean educational attainment for individuals aged 15 and older, and per capita income adjusted for inequality. The 204 countries were categorized into five levels of socio-economic development: low (<0.46), medium-low (0.46-0.60), medium (0.61-0.69), medium-high (0.70-0.81), and high (>0.81) (20, 21). These classifications allow for a systematic examination of the relationship between health outcomes and socio-economic development, with higher SDI scores indicating a higher socio-economic status.

### 2.2 Statistical analysis

To accurately measure the trends in mortality and DALYs attributable to tobacco for LOC diseases, the estimated annual percent change (EAPC) was calculated for the period from 1990 to 2021. The EAPC was computed by fitting a linear regression model to the natural logarithm of the rates with time as the independent variable. Specifically, the natural logarithm of each observed value was fitted to a straight line, and the slope of this line was calculated: y = α + βx + ϵ, where EAPC = 100 × (exp(β) − 1). In this equation, x represents the year, y represents the natural logarithm of the mortality rate, α is the intercept, β is the slope, ϵ is the random error, and exp denotes the exponential function. If the EAPC and its lower limit of the 95% confidence interval (CI) are both positive, it indicates an increasing burden. Conversely, if the EAPC and its upper limit of the 95% CI are both negative, it signifies a decreasing trend in the disease burden.

### 2.3 Age–period–cohort model

The age-period-cohort (APC) model comprehensively accounts for the potential effects of age (diagnosis age), period (diagnosis year), and cohort (birth year) on LOC diseases attributable to tobacco. The cohort effect is calculated using the formula: “cohort = period - age.” In the APC model, the results provide several indicators, including local drift, longitudinal age curve, period ratio rate (RR), and cohort relative risk. Local drift represents the log-linear trend within each age group, stratified by period and cohort. The longitudinal age curve represents the expected age-specific rates for the reference cohort, adjusted for period effects. The period (or cohort) RR represents the relative risk for different periods (or cohorts) in the population, adjusted for age and cohort (or period). An RR > 1 indicates a higher relative risk of tobacco-attributable LOC compared to the reference cohort, while an RR < 1 indicates a lower relative risk.

### 2.4 BAPC model projection

The Bayesian Age-Period-Cohort (BAPC) model, based on a Bayesian framework, incorporates age, period, and birth cohort to forecast future trends in indicators such as mortality and DALYs attributable to tobacco for LOC diseases. To model the evolving nature of these effects over time, a second-order random walk is employed for smoothing, which yields more accurate posterior predictive distributions. A key advantage of the BAPC model is its use of the Integrated Nested Laplace Approximation (INLA) method to approximate the marginal posterior distributions. This approach effectively circumvents challenges commonly associated with Markov Chain Monte Carlo (MCMC) techniques, such as issues with mixing and convergence, while maintaining high computational efficiency. The model’s flexibility and robustness in handling time-series data make it particularly well-suited for long-term disease burden forecasting (22). In this study, we predicted the changes in ASMR and ASDR for tobacco-attributable LOC, with projections extending to the year 2050.

### 2.5 Decomposition analysis

A decomposition analysis is a statistical method that breaks down the change in disease burden over time into the contributions of key driving factors. These key drivers include population growth, population aging, and epidemiological changes. By applying the Das Gupta decomposition method, the changes in mortality and DALYs attributable to tobacco for LOC diseases from 1990 to 2021 were analyzed, and the contributions of population aging, population growth, and changes in epidemiological trends were quantified (23). The Das Gupta method represents an extension and refinement of traditional decomposition analysis. Its primary function is to compare the differences between two populations across multiple dimensions and to further quantify the relative contribution of each dimensional difference to the overall gap. Compared with the traditional Kitagawa decomposition method, the Das Gupta method offers two key advantages. First, it effectively eliminates the confounding effects of interactions between different factors on the decomposition results. Second, by holding other factors constant, it avoids bias in the results that can arise from the order in which variables are entered into the model (24).

### 2.6 Cross-country inequality analysis

The slope index of inequality (SII) and the concentration index (CI) were used to assess cross-national inequalities in mortality and DALYs attributable to tobacco for LOC diseases between 1990 and 2021, with the SDI serving as a proxy for socio-economic status. The SII measures absolute inequality, where the absolute value of the SII represents the absolute difference in mortality and DALY rates between countries with lower SDI and those with higher SDI. An increasing SII value indicates widening inequality between the two groups over time, whereas a decreasing SII value suggests a narrowing gap.

The CI is a measure of relative inequality, reflecting the degree to which inequality is concentrated among either the disadvantaged or advantaged groups. The CI value ranges between -1 and 1, with a larger absolute value indicating a higher concentration of inequality. A positive value signifies that inequality is concentrated in countries with higher SDI, while a negative value indicates concentration in countries with lower SDI. The methodological details of the SII and CI have been extensively documented in the relevant literature (25, 26).

### 2.7 Frontier analysis

Frontier analysis identified leading countries or regions with the lowest burden of LOC attributable to tobacco relative to their SDI levels, highlighting areas driving progress. “Effective difference” was defined as the distance from the frontier, reflecting the gap between the observed LOC burden and the potentially achievable burden based on SDI. This gap can be minimized or eliminated through the optimization of socio-demographic resources.

All statistical analyses were performed using R 4.4.3, with two-sided p-values < 0.05 considered statistically significant to ensure the reliability and meaningfulness of the research findings.

## 3 Results

### 3.1 Global and regional burden of LOC attributable to tobacco

**Figure 1:**
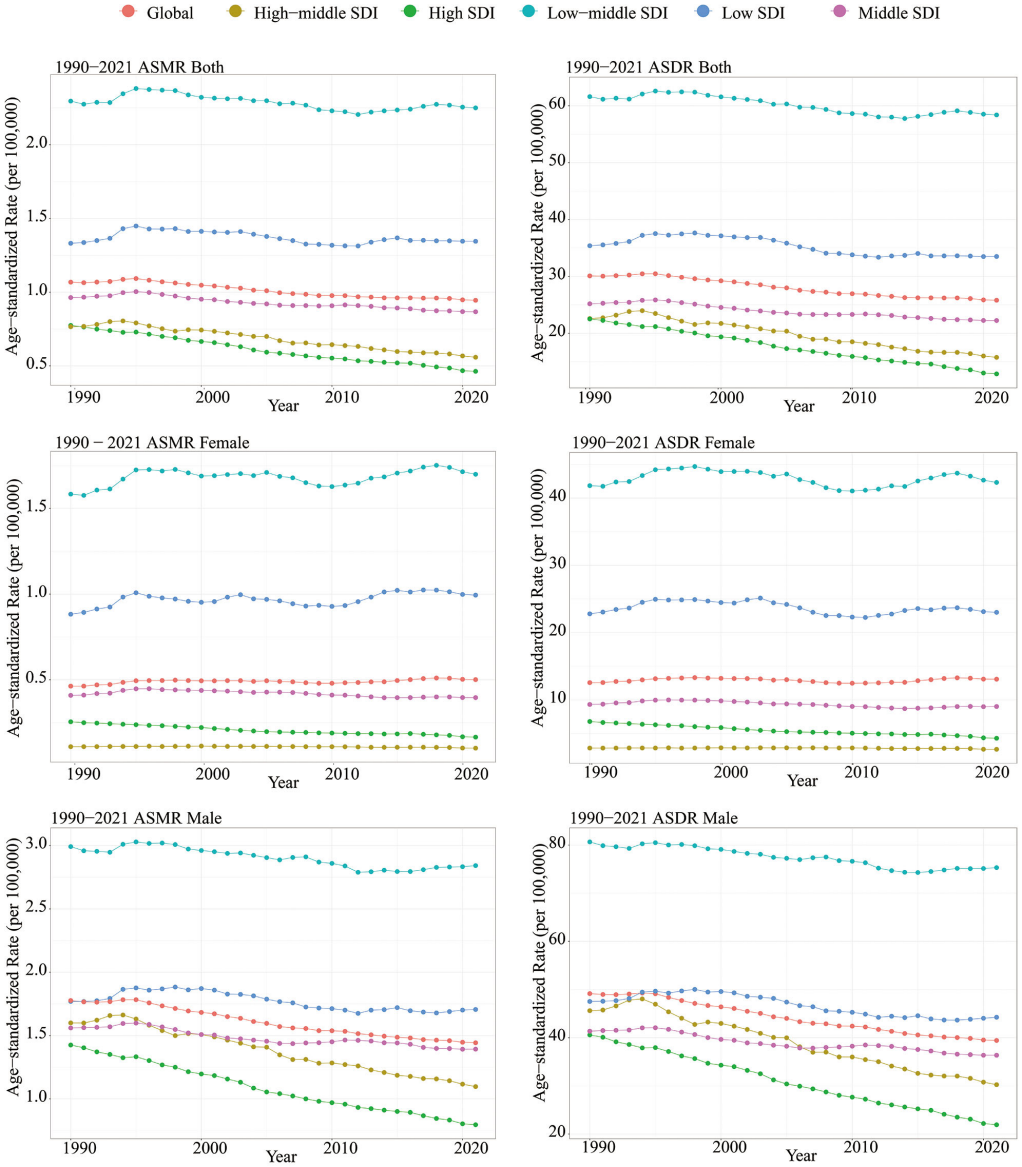
Between 1990 and 2021, age-standardized mortality rate and age-standardized DALY rate of lip and oral cancer caused by tobacco classified according to SDI.

Globally, the number of deaths from LOC attributable to tobacco increased sharply from 42,705 in 1990 to 81,947 in 2021. The ASMR for LOC due to tobacco was 1.07 per 100,000 (95% UI: 0.87 - 1.26) in 1990 and decreased to 0.95 per 100,000 (95% UI: 0.76 - 1.13) in 2021, with an EAPC of -0.49 (95% CI: -0.54 to -0.45), indicating an overall nearly stable trend (**Table 1**). Similarly, the number of DALYs attributable to tobacco for LOC increased from 1,260,941 in 1990 to 2,260,335 in 2021. The ASDR was 30.09 per 100,000 (95% UI: 24.52 - 35.52) in 1990 and showed a slight decrease to 25.80 per 100,000 (95% UI: 20.76 - 30.84) in 2021, with an EAPC of -0.61 (95% CI: -0.65 to -0.56) (**Table 2**, **Figure 1**).

**Table 1:** Age-standardized mortality rate and EAPC of lip and oral cancer caused by tobacco smoke globally and regionally from 1990 to 2021.

Location	1990		2021		EAPC (95% CI)
	Number (95% UI)	ASR (95% UI)	Number (95% UI)	ASR (95% UI)	
Global	42705 (34703 - 50564)	1.07 (0.86 - 1.26)	81947 (65940 - 98083)	0.95 (0.76 - 1.13)	-0.49 (-0.54 to -0.45)
Socio-demographic index					
High SDI	8318 (6047 - 10549)	0.77 (0.56 - 0.98)	9263 (6331 - 12333)	0.46 (0.32 - 0.61)	-1.65 (-1.7 to -1.61)
High-middle SDI	7772 (5822 - 9560)	0.77 (0.57 - 0.95)	11134 (8073 - 14025)	0.56 (0.41 - 0.7)	-1.19 (-1.27 to -1.11)
Middle SDI	9679 (7850 - 11450)	0.96 (0.78 - 1.14)	23142 (18263 - 28258)	0.87 (0.69 - 1.06)	-0.45 (-0.5 to -0.39)
Low-middle SDI	13934 (11506 - 16597)	2.3 (1.9 - 2.72)	31863 (26333 - 37621)	2.25 (1.86 - 2.65)	-0.15 (-0.21 to -0.1)
Low SDI	2960 (2360 - 3631)	1.33 (1.07 - 1.62)	6491 (5053 - 7887)	1.34 (1.06 - 1.62)	-0.16 (-0.25 to -0.06)
Region					
Andean Latin America	23 (16 - 32)	0.12 (0.08 - 0.17)	48 (31 - 69)	0.08 (0.05 - 0.12)	-1.01 (-1.11 to -0.9)
Australasia	136 (93 - 179)	0.58 (0.4 - 0.77)	130 (84 - 189)	0.25 (0.16 - 0.36)	-2.69 (-2.98 to -2.4)
Caribbean	189 (132 - 248)	0.74 (0.52 - 0.98)	298 (203 - 406)	0.55 (0.37 - 0.75)	-0.83 (-0.9 to -0.76)
Central Asia	292 (212 - 367)	0.59 (0.43 - 0.75)	358 (255 - 463)	0.42 (0.29 - 0.54)	-1 (-1.15 to -0.84)
Central Europe	1726 (1283 - 2163)	1.15 (0.86 - 1.45)	2076 (1444 - 2687)	1.03 (0.72 - 1.32)	-0.43 (-0.55 to -0.31)
Central Latin America	228 (161 - 296)	0.3 (0.21 - 0.39)	318 (217 - 430)	0.13 (0.09 - 0.18)	-2.95 (-3.08 to -2.82)
Central Sub-Saharan Africa	54 (37 - 76)	0.24 (0.17 - 0.33)	128 (88 - 177)	0.24 (0.16 - 0.34)	-0.08 (-0.19 to 0.04)
East Asia	4125 (3064 - 5229)	0.49 (0.36 - 0.62)	11427 (7858 - 15474)	0.52 (0.36 - 0.7)	0.45 (0.27 to 0.63)
Eastern Europe	2934 (2213 - 3554)	1.03 (0.78 - 1.25)	3313 (2398 - 4137)	0.98 (0.71 - 1.22)	-0.67 (-0.92 to -0.41)
Eastern Sub-Saharan Africa	333 (257 - 422)	0.46 (0.36 - 0.59)	632 (443 - 826)	0.38 (0.27 - 0.5)	-0.74 (-0.79 to -0.69)
High-income Asia Pacific	805 (609 - 990)	0.4 (0.3 - 0.49)	1427 (975 - 1925)	0.3 (0.21 - 0.4)	-1.38 (-1.75 to -1)
High-income North America	2793 (2032 - 3567)	0.83 (0.6 - 1.05)	2690 (1869 - 3618)	0.41 (0.29 - 0.55)	-2.21 (-2.37 to -2.06)
North Africa and Middle East	377 (264 - 495)	0.23 (0.16 - 0.3)	834 (587 - 1085)	0.19 (0.13 - 0.24)	-0.78 (-0.84 to -0.71)
Oceania	11 (7 - 15)	0.34 (0.22 - 0.47)	30 (19 - 40)	0.34 (0.23 - 0.47)	0.12 (0.04 to 0.2)
South Asia	19542 (16243 - 23381)	3.41 (2.83 - 4.07)	46348 (37885 - 55064)	3.18 (2.61 - 3.75)	-0.39 (-0.47 to -0.31)
Southeast Asia	2504 (1993 - 3030)	1.09 (0.88 - 1.31)	5792 (4527 - 7088)	0.96 (0.75 - 1.18)	-0.59 (-0.67 to -0.51)
Southern Latin America	253 (174 - 330)	0.54 (0.37 - 0.71)	253 (169 - 347)	0.3 (0.2 - 0.41)	-1.69 (-1.97 to -1.4)
Southern Sub-Saharan Africa	271 (169 - 375)	1.02 (0.64 - 1.39)	385 (282 - 489)	0.67 (0.49 - 0.85)	-1.56 (-1.74 to -1.38)
Tropical Latin America	964 (700 - 1226)	1.06 (0.76 - 1.35)	1349 (921 - 1823)	0.52 (0.35 - 0.7)	-2.51 (-2.66 to -2.36)
Western Europe	5062 (3602 - 6443)	0.94 (0.67 - 1.19)	3897 (2587 - 5285)	0.45 (0.31 - 0.61)	-2.29 (-2.34 to -2.24)
Western Sub-Saharan Africa	84 (62 - 113)	0.1 (0.07 - 0.13)	215 (152 - 293)	0.11 (0.08 - 0.16)	0.37 (0.27 to 0.46)

**Table 2:** Age-standardized DALY rate and EAPC of lip and oral cancer caused by tobacco smoke globally and regionally from 1990 to 2021.

Location	1990		2021		EAPC (95% CI)
	Number (95% UI)	ASR (95% UI)	Number (95% UI)	ASR (95% UI)	
Global	1260941 (1029660 - 1487189)	30.086 (24.519 - 35.517)	2260335 (1816976 - 2702377)	25.801 (20.758 - 30.838)	-0.61 (-0.65 to -0.56)
Socio-demographic index					
High SDI	235393 (173110 - 295464)	22.51 (16.57 - 28.2)	235715 (163392 - 306728)	12.86 (8.99 - 16.62)	-1.8 (-1.84 to -1.76)
High-middle SDI	235003 (177335 - 286604)	22.59 (17.01 - 27.57)	310642 (225596 - 388574)	15.76 (11.45 - 19.69)	-1.38 (-1.47 to -1.29)
Middle SDI	283590 (230811 - 336895)	25.18 (20.45 - 29.81)	626360 (491920 - 774685)	22.24 (17.51 - 27.42)	-0.51 (-0.56 to -0.46)
Low-middle SDI	416500 (345584 - 497666)	61.61 (50.95 - 73.78)	899503 (736687 - 1063930)	58.39 (48.09 - 69.09)	-0.25 (-0.3 to -0.2)
Low SDI	89172 (70585 - 110326)	35.39 (28.21 - 43.55)	186613 (144216 - 229614)	33.5 (26.11 - 40.95)	-0.39 (-0.49 to -0.29)
Region					
Andean Latin America	601 (419 - 827)	2.84 (1.98 - 3.94)	1163 (776 - 1651)	1.94 (1.29 - 2.76)	-1.09 (-1.2 to -0.99)
Australasia	3767 (2598 - 4904)	16.49 (11.41 - 21.46)	3374 (2230 - 4816)	7.02 (4.65 - 9.96)	-2.72 (-2.98 to -2.45)
Caribbean	4880 (3484 - 6345)	18.63 (13.26 - 24.27)	7665 (5320 - 10387)	14.08 (9.76 - 19.11)	-0.77 (-0.84 to -0.7)
Central Asia	9075 (6616 - 11400)	17.84 (12.99 - 22.42)	10697 (7658 - 13695)	11.59 (8.26 - 14.92)	-1.31 (-1.45 to -1.16)
Central Europe	54042 (40456 - 67138)	36.29 (27.14 - 45.07)	59324 (41673 - 75707)	31.17 (21.98 - 39.58)	-0.6 (-0.75 to -0.45)
Central Latin America	5655 (4052 - 7264)	6.74 (4.8 - 8.69)	7596 (5236 - 10197)	2.99 (2.06 - 4.01)	-2.94 (-3.07 to -2.8)
Central Sub-Saharan Africa	1621 (1104 - 2328)	6.41 (4.39 - 9.07)	3883 (2652 - 5390)	6.13 (4.2 - 8.51)	-0.16 (-0.27 to -0.04)
East Asia	118166 (87740 - 150153)	12.62 (9.35 - 16.03)	298197 (205298 - 402632)	13.17 (9.09 - 17.76)	0.38 (0.21 to 0.56)
Eastern Europe	93220 (70651 - 112164)	32.93 (24.9 - 39.68)	101811 (74326 - 126738)	31.43 (23.03 - 39.05)	-0.71 (-0.96 to -0.46)
Eastern Sub-Saharan Africa	9728 (7464 - 12274)	11.97 (9.24 - 15.19)	18614 (13029 - 24372)	9.89 (6.94 - 12.93)	-0.77 (-0.82 to -0.72)
High-income Asia Pacific	22219 (16866 - 27104)	10.7 (8.11 - 13.07)	29684 (20727 - 39174)	7.62 (5.38 - 9.95)	-1.59 (-1.98 to -1.2)
High-income North America	76314 (55789 - 96029)	23.67 (17.35 - 29.69)	68251 (47799 - 89974)	11.13 (7.83 - 14.58)	-2.39 (-2.54 to -2.25)
North Africa and Middle East	11007 (7751 - 14425)	5.97 (4.18 - 7.85)	23764 (16773 - 30686)	4.75 (3.35 - 6.17)	-0.87 (-0.94 to -0.8)
Oceania	366 (234 - 504)	10.06 (6.42 - 13.87)	1037 (682 - 1443)	10.6 (6.99 - 14.56)	0.28 (0.19 to 0.36)
South Asia	593608 (489409 - 712800)	91.61 (75.97 - 109.56)	1319404 (1065838 - 1574944)	83.42 (67.91 - 99.24)	-0.45 (-0.51 to -0.38)
Southeast Asia	66030 (52347 - 80442)	25.27 (20.12 - 30.69)	146250 (113168 - 181613)	21.64 (16.93 - 26.71)	-0.66 (-0.72 to -0.6)
Southern Latin America	7595 (5293 - 9747)	16.17 (11.27 - 20.76)	7038 (4810 - 9455)	8.45 (5.78 - 11.31)	-1.89 (-2.18 to -1.6)
Southern Sub-Saharan Africa	7988 (5008 - 11064)	27.52 (17.15 - 38.2)	11124 (8005 - 14352)	17.76 (12.95 - 22.74)	-1.64 (-1.81 to -1.47)
Tropical Latin America	28288 (20542 - 35815)	28.6 (20.74 - 36.34)	36171 (24560 - 48137)	13.63 (9.26 - 18.17)	-2.68 (-2.86 to -2.51)
Western Europe	144378 (104456 - 181638)	28.13 (20.38 - 35.34)	99091 (66946 - 131949)	12.78 (8.72 - 16.87)	-2.53 (-2.57 to -2.48)
Western Sub-Saharan Africa	2395 (1742 - 3211)	2.56 (1.88 - 3.42)	6198 (4287 - 8521)	2.84 (1.99 - 3.88)	0.25 (0.14 to 0.35)

All SDI quantities demonstrated a declining trend in both ASMR and ASDR. The highest SDI quintile observed the most significant reductions in ASMR and ASDR, with EAPCs of -1.65 (95% CI: -1.70 to -1.61) and -1.80 (95% CI: -1.84 to -1.76), respectively. Over the past three decades, SDI quintiles with lower levels exhibited slight decline in ASMR and ASDR for tobacco-attributable LOC (**Table 1**). Among these, the low-middle SDI quintile had the highest burden. In 2021, the ASMR was 2.25 per 100,000 (95% UI: 1.86 - 2.65), and the ASDR was 58.39 per 100,000 (95% UI: 48.09 - 69.09) (**Table 2**, **Figure 1**).

Among the 21 GBD regions in 2021, South Asia had the highest ASMR and ASDR, at 3.18 (95% CI: 2.61 - 3.75) and 83.42 (95% CI: 67.91 - 99.24) per 100,000, respectively. Additionally, only East Asia, Oceania, and Western Sub-Saharan Africa showed stable or slightly increasing trends in ASMR and ASDR, while the remaining regions exhibited declining trends. The most significant declines in ASMR were observed in Central Latin America, Australasia, and Tropical Latin America, with EAPCs of -2.95 (95% CI: -3.08 to -2.82), -2.69 (95% CI: -2.98 to -2.40), and -2.51 (95% CI: -2.66 to -2.36), respectively. Similarly, the most pronounced declines in ASDR were noted in these regions, with EAPCs of -2.94 (95% CI: -3.07 to -2.80), -2.72 (95% CI: -2.98 to -2.45), and -2.68 (95% CI: -2.86 to -2.51), respectively (**Tables 1**, **2**).

### 3.2 Differences in the burden of LOC attributable to tobacco in 204 countries and regions

**Figure 2:**
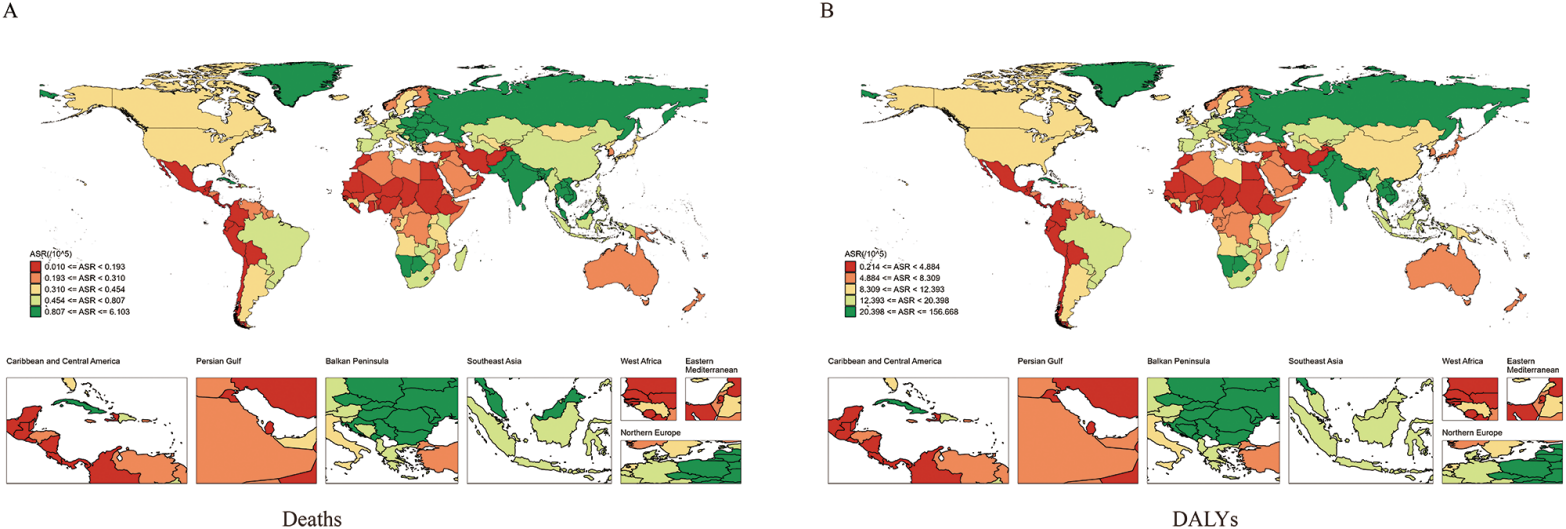
Global distribution map of age-standardized mortality rate **(A)** and age-standardized DALY rate **(B)** of lip and oral cancer caused by tobacco between 1990 and 2021.

Between 1990 and 2021, significant differences in the burden of LOC attributable to tobacco were observed at the national level. In 2021, the highest ASMRs were recorded in Pakistan (6.10/100,000), Palau (4.57/100,000), and Bangladesh (2.97/100,000). The lowest ASMR was observed in Sao Tome and Principe (**Figure 2A**; **Supplementary Table 1**). The largest increase in ASMR was seen in Cabo Verde (EAPC = 7.79, 95% CI: 5.22 to 10.42), while the most significant decrease occurred in Colombia (EAPC = -4.04, 95% CI: -4.26 to -3.82) (**Supplementary Figure S1A**, **Supplementary Table 1**).

In 2021, the highest ASDRs were recorded in Pakistan (156.67/100,000), Palau (147.26/100,000), and India (76.89/100,000) (**Figure 2B**; **Supplementary Table 1**). The most significant annual increase in ASDR was observed in Cabo Verde (EAPC = 7.5, 95% CI: 4.94 to 10.13), while the most significant decrease was seen in Colombia (EAPC = -4.17, 95% CI: -4.4 to -3.93) (**Supplementary Figure S1B**, **Supplementary Table 1**).

### 3.3 Association between tobacco-attributable LOC burden and SDI in GBD regions

**Figure 3:**
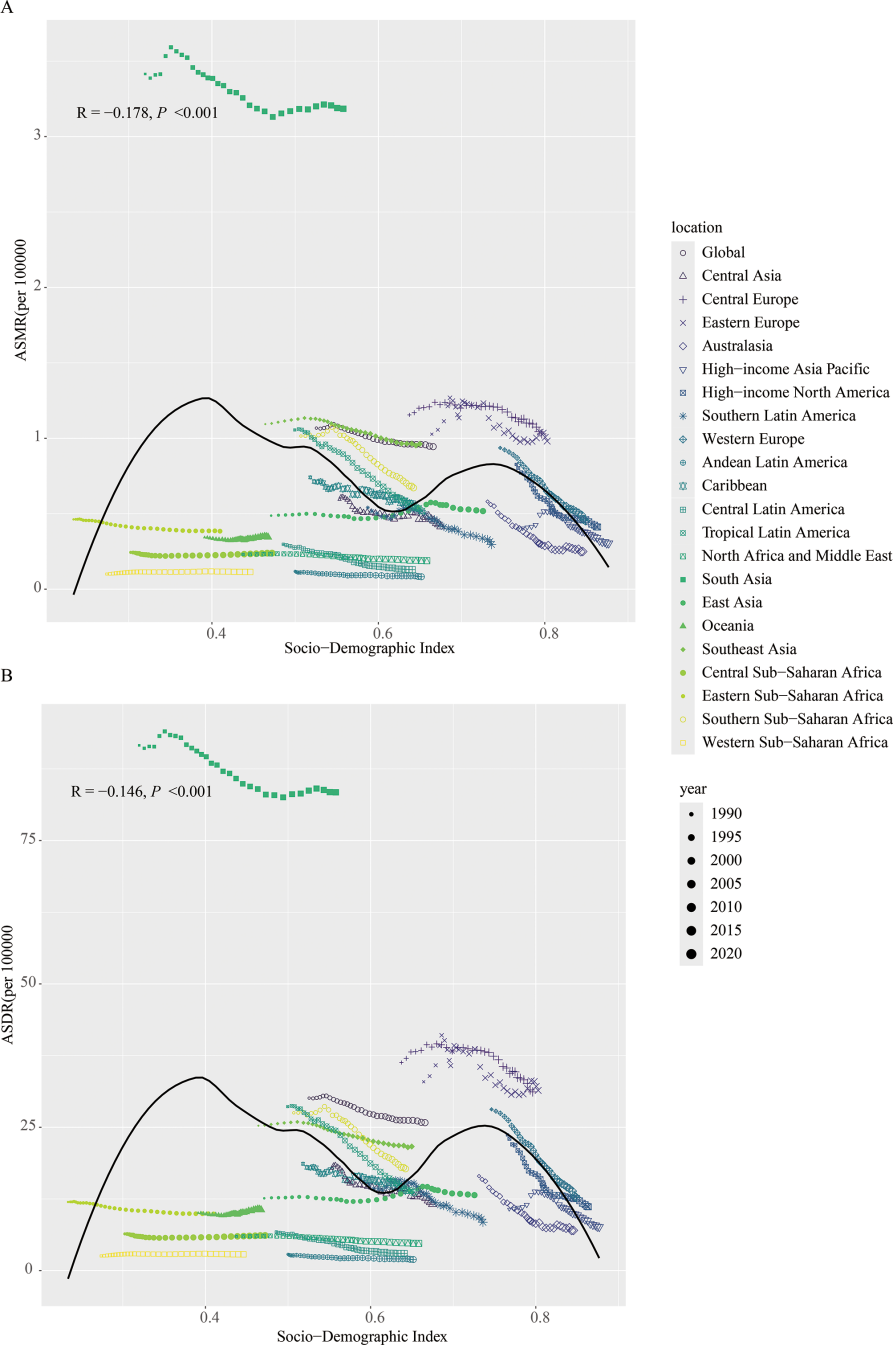
Between 1990 and 2021, the correlation between age-standardized mortality rate **(A)** and age-standardized DALY rate **(B)** of lip and oral cancer caused by tobacco with SDI.

The relationship between ASMR (R = -0.178, *P* < 0.001)/ASDR (R = -0.146, *P* < 0.001) and SDI showed an “M”-shaped pattern. Specifically, ASMR and ASDR increased with SDI when SDI was below 0.4, decreased between SDI 0.4 and 0.6, increased again between SDI 0.6 and 0.75, and decreased when SDI exceeded 0.75 (**Figure 3**).

### 3.4 Impact of APC on mortality and DALYs

**Figure 4:**
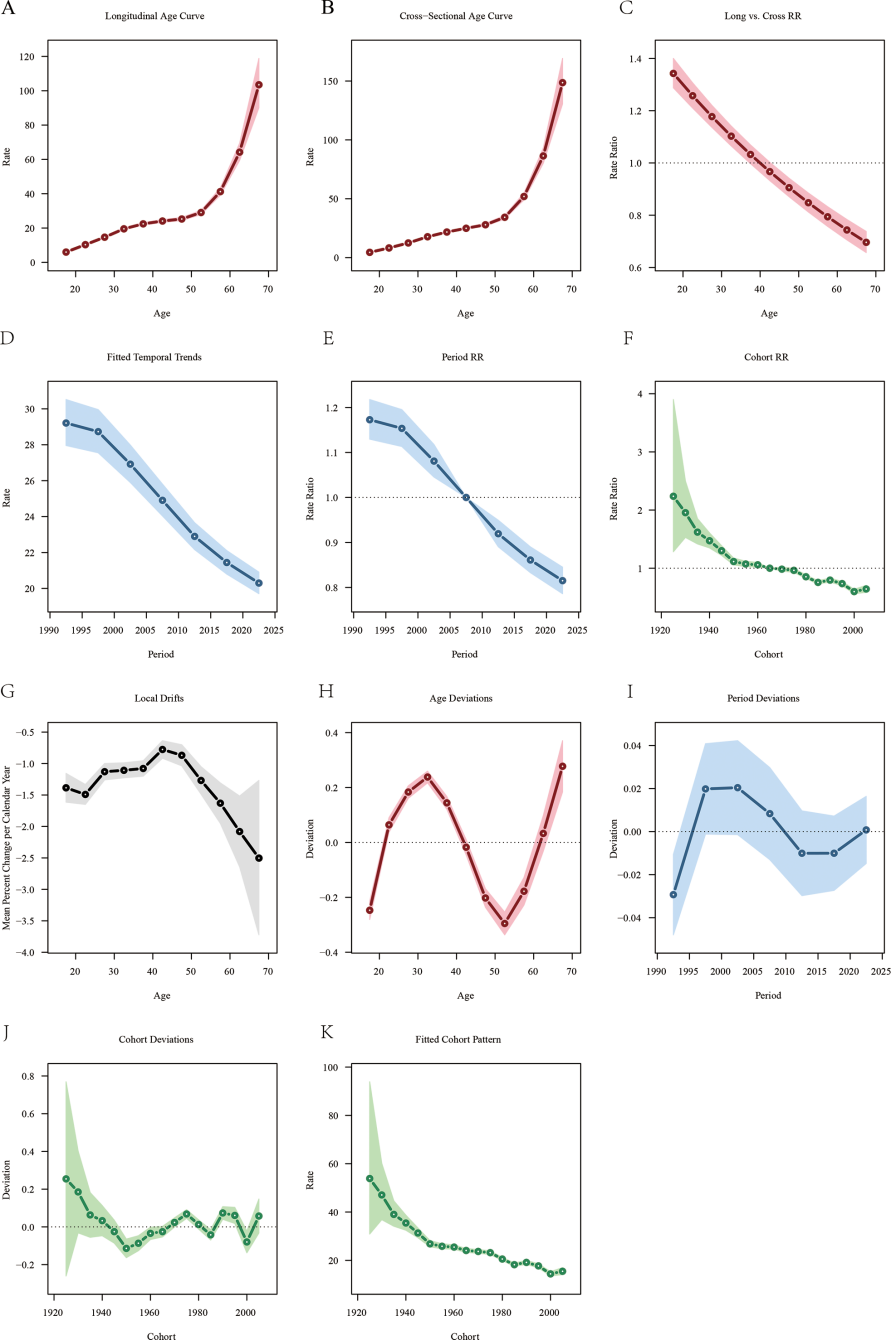
Age-period-cohort analysis of lip and oral cancer mortality attributable to tobacco from 1990 to 2021. **(A)** Longitudinal age-specific mortality rate curves for liver cancer; **(B)** Cross-sectional age-specific mortality rate curves for lip and oral cancer; **(C)** Comparison of longitudinal and cross-sectional rate ratios; **(D)** Fitted time trends for lip and oral cancer mortality rates; **(E)** Changes in period rate ratios over time; **(F)** Cohort rate ratios by birth cohort; **(G)** Local drift in lip and oral cancer mortality rates by age; **(H)** Deviation between age and the fitted model; **(I)** Deviation between period and the fitted model; **(J)** Deviation between cohort and the fitted model; **(K)** Fitted cohort model for lip and oral cancer mortality rates.

**Figure 4** illustrates the effects of age, period, and cohort on the mortality of LOC. The longitudinal age curve, as shown in **Figure 4A**, indicates that the mortality rate of LOC attributable to tobacco increases with age. Notably, a significant increase in tobacco-attributable mortality began at age 52.5. For the period effect, the RR of ASMR showed a declining trend over time, decreasing from 1.173 in 1992 to 0.815 in 2021 (**Figure 4E**). For the cohort effect, the mortality rate of LOC attributable to tobacco was lower in later-born cohorts compared to earlier-born cohorts. The RR value decreased from 2.237 (95% CI: 1.283 - 3.900) for the 1925 birth cohort to 0.643 (95% CI: 0.585 - 0.706) for the 2005 birth cohort (**Figure 4F**). Additionally, in this study, the local drift value was < 0, indicating an overall declining trend in tobacco-related ASMR (**Figure 4G**).

The trends for age, period, and cohort effects on DALYs for LOC were opposite. The longitudinal age curve showed a decreasing trend in DALYs for LOC in the age group under 37.5 years, while DALYs for those 37.5 years and older gradually stabilized (**Supplementary Figure S2A**). The overall trend in DALYs for LOC across different periods followed a V-shape, with the RR value increasing from 1.038 in 1992 to 1.046 in 1997, then gradually decreasing to 0.992 in 2012, and subsequently rising to 1.046 in 2022 (**Supplementary Figure S2E**). Earlier-born cohorts had higher DALYs for LOC [RRcohort (1940) = 0.941, 95% CI: 0.731 - 1.212], whereas more recent-born cohorts had lower DALYs [RRcohort (2005) = 0.838, 95% CI: 0.825 - 0.852] (**Supplementary Figure S2F**). Additionally, the local drift value in this study indicated an overall increasing trend in tobacco-related DALYs (**Supplementary Figure S2G**).

### 3.5 BAPC model

**Figure 5:**
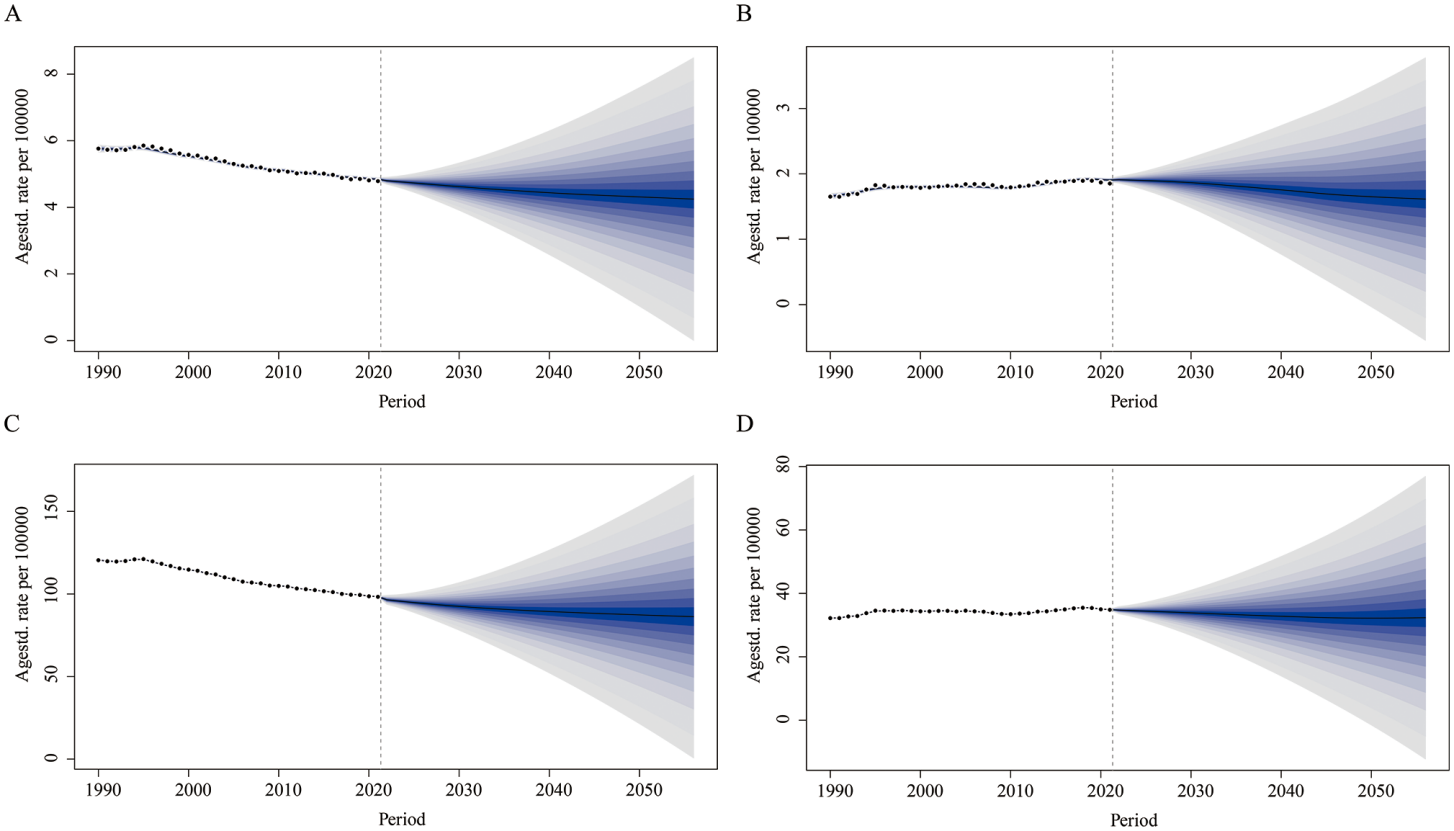
Predicted global trends in age-standardized mortality rate and age-standardized DALY rate for lip and oral cancer attributable to tobacco from 2021 to 2050. **(A)** Predicting the trend of age-standardized mortality rates for males; **(B)** Predicting the trend of age-standardized mortality rates for females; **(C)** Predicting the trend of age-standardized DALY rates for males; **(D)** Predicting the trend of age-standardized DALY rates for females.

Between 1990 and 2021, the ASMR for LOC attributable to tobacco showed a continuous decline globally for both males and females. It is projected that global mortality will further decrease by 2050. The male ASMR is expected to decline from 4.85 per 100,000 in 2021 to 4.31 per 100,000 in 2050 (**Figure 5A**), while the female ASMR will slightly decrease from 1.91 per 100,000 in 2021 to 1.65 per 100,000 in 2050 (**Figure 5B**). Similarly, DALYs have been decreasing since 1990 and are projected to continue declining by 2050. Notably, the reduction is more pronounced in males, falling from 98.41 per 100,000 in 2021 to 87.32 per 100,000 in 2050 (**Figure 5C**). In females, the decline is more modest, decreasing from 34.91 per 100,000 in 2021 to 32.20 per 100,000 in 2050 (**Figure 5D**).

### 3.6 Global and SDI regional decomposition analysis

**Figure 6:**
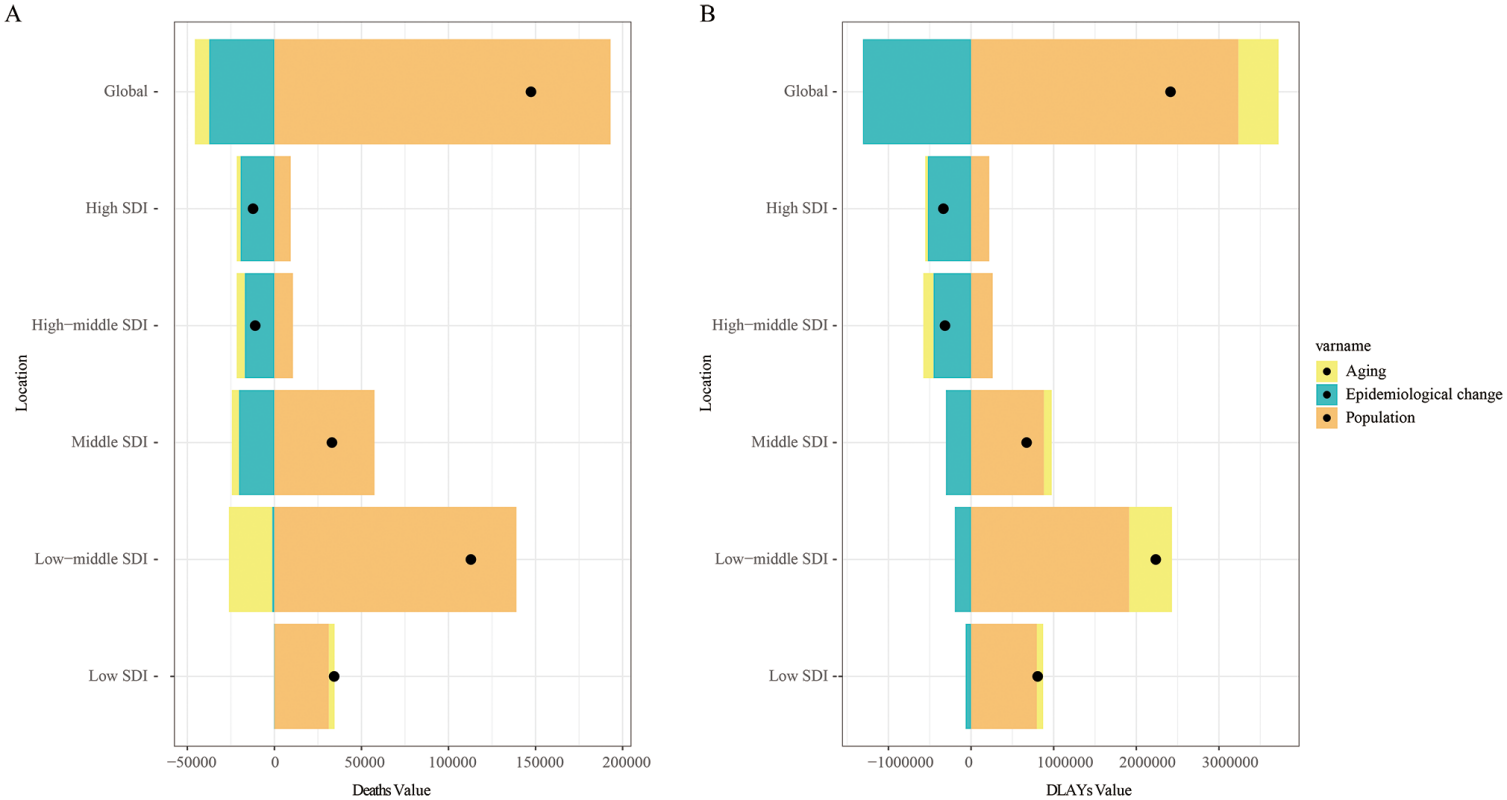
Between 1990 and 2021, the determinants of aging, population growth, and epidemiological changes in lip and oral cancer caused by tobacco globally and in various SDI regions. Black dots indicate the net total change in disease burden. **(A)** Number of deaths; **(B)** Disability-adjusted life years.

A decomposition analysis examined the contributions of population aging, population growth, and epidemiological changes to the mortality and DALYs attributable to tobacco for LOC between 1990 and 2021. The increase in global LOC mortality was primarily attributed to population growth (131.02%), while the decrease in global mortality was mainly due to epidemiological changes (-25.43%). The most pronounced upward trend in LOC mortality was observed in the low-middle SDI regions, where population growth (123.18%) contributed the most. In contrast, the downward trend in mortality in high SDI regions (-158.65%) and high-middle SDI regions (-154.24%) was primarily driven by epidemiological changes (**Figure 6A**). Among the 21 GBD regions, the significant increase in LOC mortality in South Asia was largely attributed to population growth (89.69%) (**Supplementary Figure S3A**).

Similar results were observed for DALYs. The increase in global DALYs due to tobacco-attributable LOC was primarily attributed to population growth (134%), followed by population aging (20.2%). Notably, the most significant increase in DALYs occurred in the low-middle SDI regions, largely driven by population growth (85.54%) (**Figure 6B**). Among the 21 GBD regions, the increase in DALYs for LOC in South Asia was similarly attributed to population growth (101.46%) (**Supplementary Figure S3B**).

### 3.7 Transnational inequality analysis of LOC attributable to tobacco

**Figure 7:**
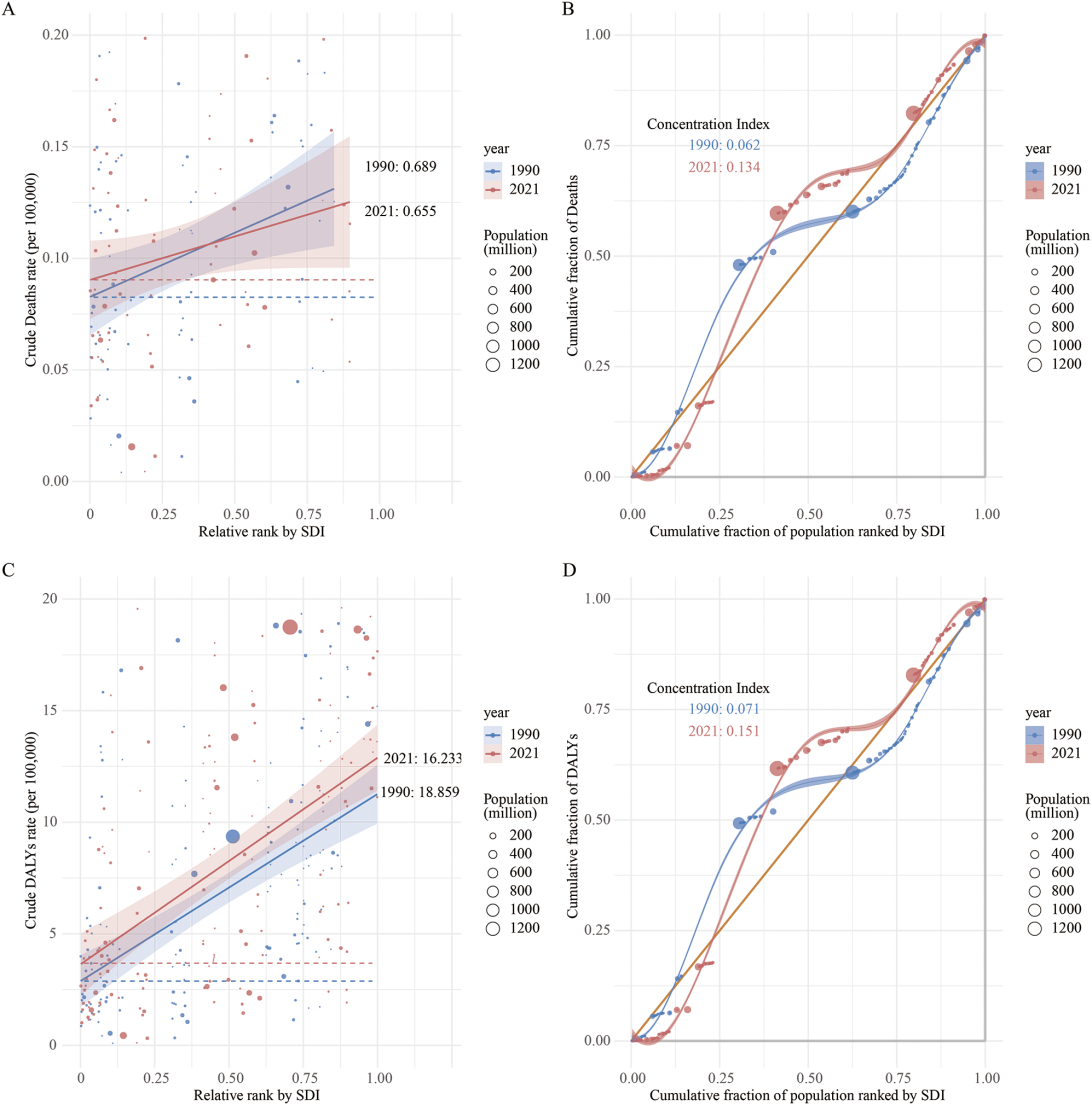
Regression curves and concentration curves for health inequalities in lip and oral cancer caused by tobacco in 1990 and 2021: **(A)** Regression curve for health inequalities in mortality; **(B)** Concentration curve for mortality; **(C)** Regression curve for health inequalities in disability-adjusted life years; **(D)** Concentration curve for DALY.

In 1990 and 2021, the SII for the ASMR of LOC attributable to tobacco across countries and regions with different SDI levels was 0.689 (95% CI: 0.534 to 0.844) and 0.655 (95% CI: 0.500 to 0.810), respectively. Moreover, an upward trend in ASMR was observed with increasing SDI, indicating that regions with higher SDI levels tend to be associated with higher ASMRs for LOC. Additionally, the inequality in the burden of LOC between high-SDI and low-SDI countries decreased in 2021 (**Figure 7A**). Similarly, the CI for ASMR in 2021 was 0.134 (95% CI: 0.043 to 0.233), which increased compared to 1990 (CI: 0.062, 95% CI: -0.026 to 0.144), suggesting an increase in inequality among higher SDI countries (**Figure 7B**).

Regarding DALYs, the SII for tobacco-attributable LOC DALYs in 2021 was 16.233 (95% CI: 12.040 to 20.427), compared to 18.859 (95% CI: 14.456 to 23.262) in 1990. This indicates a reduction in inequality between higher and lower SDI countries over time (**Figure 7C**). Furthermore, the CI also showed an increase in relative inequality among higher SDI countries over time, rising from 0.071 (95% CI: -0.018 to 0.148) in 1990 to 0.151 (95% CI: 0.055 to 0.249) in 2021 (**Figure 7D**).

### 3.8 Frontier analysis of LOC attributable to tobacco

**Figure 8:**
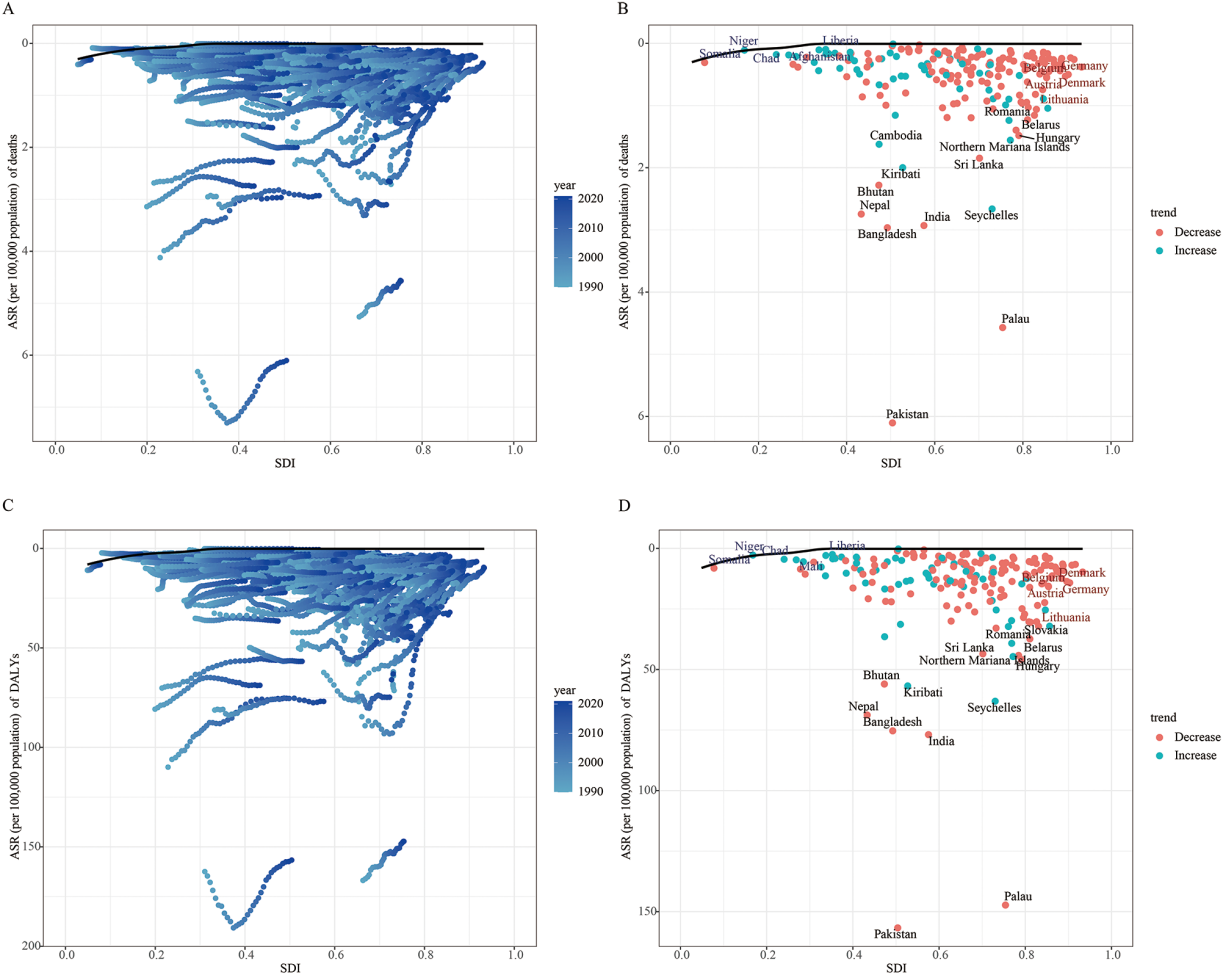
Frontier analysis of age-standardized mortality rate **(A, B)** and age-standardized DALY rate **(C, D)** based on SDI and lip and oral cancer attributable to tobacco in 2021.

**Figure 8** illustrates the unrealized health gains for LOC attributable to tobacco in countries with varying levels of development between 1990 and 2021. The effective gap widened gradually with socioeconomic and demographic development, indicating that some middle SDI countries possess greater potential for burden improvement (**Figure 8A**). In the frontier analysis of mortality attributable to tobacco-related LOC, the top 15 countries with the largest effective gaps, compared to the black boundary line, are marked in black font, including Pakistan, Palau, Bangladesh, India, Seychelles, Nepal, Bhutan, Kiribati, Sri Lanka, and Cambodia. Frontier countries with low SDI (< 0.5) and low effective gaps are marked in blue font, including Somalia, Niger, Chad, Afghanistan, and Liberia. Frontier countries and regions with high SDI (> 0.5) and effective gaps are marked in red font, specifically Belgium, Germany, Austria, Denmark, and Lithuania. Red dots represent an increase in the ASMR for tobacco-attributable LOC from 1990 to 2021, while blue dots represent a decrease in the ASMR over the same period (**Figure 8B**).

In the frontier analysis for DALYs attributable to tobacco-related LOC, some middle SDI countries again showed greater potential for burden improvement (**Figure 8C**). The top 15 countries with the largest effective gaps were Pakistan, Palau, India, Bangladesh, Seychelles, Nepal, Kiribati, Bhutan, Northern Mariana Islands, and Hungary. Frontier countries with low SDI (<0.5) and low effective gaps included Somalia, Niger, Chad, Mali, and Liberia. Frontier countries and regions with high SDI (> 0.5) and effective gaps were Denmark, Belgium, Germany, Austria, and Lithuania (**Figure 8D**).

## 4 Discussion

This study is the first to quantify the cross-national inequalities in the burden of LOC attributable to tobacco, as well as their temporal trends, from 1990 to 2021. Globally, the number of deaths and DALYs from LOC cancer attributable to tobacco increased sharply between 1990 and 2021, with this absolute growth primarily driven by population expansion. However, the ASMR and ASDR showed a slight downward trend, which is associated with epidemiological changes. The burden of LOC cancer was most severe in the low-middle SDI regions, while the ASMR and ASDR decreased most significantly in high SDI regions. In East Asia, Oceania, and West Sub-Saharan Africa, the ASMR/ASDR did not decline significantly and even slightly increased. The SII indicated a narrowing gap in the burden between high SDI and low SDI countries. Conversely, the CI reflected an increase in inequality within high SDI countries. Mortality rates showed a significant increase with age, particularly after the age of 52, although more recent birth cohort exhibited lower risk. Projections suggest that the ASMR and DALYs for LOC cancer attributable to tobacco will continue to decrease globally.

We found that over the past three decades, despite a slight global decline in the burden of LOC attributable to tobacco, the regions with the lowest and middle SDI levels still exhibited the highest ASMR and ASDR, consistent with previous research findings (17, 27). Miranda-Filho et al. observed that globally, parts of South and Central Asia and certain regions in Oceania had the highest incidence rates of LOC, with India bearing the heaviest burden (28). Our study further revealed that in South Asia, both the ASMR and ASDR for LOC attributable to tobacco were the highest. This is associated with high prevalence rates of tobacco use, inadequate enforcement of tobacco control policies, and insufficient healthcare resources in these regions (29).

In Southeast Asian countries, the high burden of LOC is also linked to the use of smokeless tobacco and areca nut chewing, which are common risk factors in these regions (30, 31). High levels of nitrosamines in smokeless tobacco are specific carcinogens that significantly increase the risk of developing LOC (32). Although many low- and middle-income countries have implemented the World Health Organization (WHO) Framework Convention on Tobacco Control, difficulties in implementation have prevented them from achieving their intended goals (33). These difficulties include limited resources, weak policy enforcement, cultural barriers, and low public awareness.

Additionally, we observed that over the past 30 years, the burden of tobacco-attributable LOC has decreased in regions with higher SDI levels. This may be attributed to the implementation of comprehensive tobacco control policies and increased public health awareness (34, 35). For example, a 2019 report by the WHO highlighted the success of Brazil in tobacco control under the guidance of the framework convention (36). Higher-income regions are more likely to participate in cancer screening programs, and early detection and control of LOC have effectively reduced mortality rates (37, 38).

At the regional and national levels, we observed a slight increase in the burden of ASMR and ASDR for LOC attributable to tobacco in East Asia, Oceania, and Western Sub-Saharan Africa. Taking China as an example, although the adult smoking prevalence decreased from 28.1% in 2010 to 24.1% in 2022, the proportion of cigarette sales accounted for globally rose from 31.7% to 47.2%. Coupled with population aging, this has kept the LOC burden at a high level (19). Notably, during the study period, Pakistan bore the heaviest burden of LOC attributable to tobacco. This aligns with previous findings; a study utilizing International Agency for Research on Cancer (IARC) statistics also indicated a high burden of oral cancer in Pakistan (28). Smokeless tobacco use is a primary risk factor for LOC in Pakistan, with approximately 25.4 million adult Pakistanis consuming tobacco products in 2022, ranking the country 7th globally in terms of tobacco product users (39). Smokeless tobacco leads to lesions such as micronuclei, nuclear budding, binucleated cells, and perinuclear halos in the buccal mucosa (40). Additionally, HPV infection and genetic susceptibility also contribute to the increased burden of LOC in Pakistan (41). Therefore, strengthening smoking cessation services is crucial in areas with a heavy burden. Most importantly, alongside disseminating information about the harms of smoking, efforts must also focus on reducing the availability and accessibility of tobacco products.

We also examined the effects of age, period, and cohort on the ASMR and ASDR for LOC attributable to tobacco. Age has long been recognized as one of the primary factors associated with LOC. Our findings indicate that the mortality from tobacco-attributable LOC increases gradually with age, with the upward trend becoming more pronounced after the age of 50. In older adults, the efficiency of DNA damage repair mechanisms declines, making it easier for carcinogens in tobacco to induce gene mutations that accumulate over time, thereby increasing the risk of LOC (42). Additionally, the decline in physical and cognitive abilities in the elderly population can lead to difficulties in maintaining routine oral hygiene and reduced access to oral healthcare services, which may further exacerbate the risk of developing LOC (43). However, period risks showed a consistent decline year by year. The cohort effect revealed that individuals born later had a lower risk compared to those born earlier. BAPC projections indicate that the burden of tobacco-attributable LOC will continue to decrease. This trend can be attributed to the implementation of the Framework Convention on Tobacco Control (FCTC) and improvements in healthcare quality. For instance, China increased the cigarette excise tax from 5% to 11% since 2015, effectively curbing tobacco consumption (44). Globally, 57 countries have implemented policies targeting smokeless tobacco, resulting in a reduction of its prevalence from 4.4% to 30.3% (45). While the current trends are encouraging, sustained investment in tobacco control, healthcare systems, and treatment modalities will remain crucial for further reducing the long-term burden of tobacco-attributable LOC and improving the quality of life for affected patients.

Regarding the global burden of LOC attributable to tobacco, health inequalities have improved from 1990 to 2021, as indicated by a decrease in the SII, suggesting a more equitable distribution of the burden. However, high-income countries continue to face a disproportionately higher burden of tobacco-attributable LOC, reflecting significant disparities in global health resources and the quality of care. This may be attributed to characteristics of high SDI regions, such as comprehensive healthcare infrastructure, advanced capabilities for early diagnosis, and specialized care, which lead to more comprehensive registration of LOC mortality and disease burden statistics. A key factor is that, although high SDI countries implemented tobacco control measures earlier, the effectiveness of these policies has been uneven in some nations. Another critical factor is the social stratification of smoking rates in high SDI countries, where lower educational attainment and lower income levels are associated with higher smoking prevalence. For example, in the United States, smoking rates among impoverished populations, including the uptake of heated tobacco products, have doubled within a year (46). This phenomenon underscores the need for long-term monitoring of the effectiveness of tobacco control policies and highlights that low SDI countries, if they do not strengthen their tobacco control efforts, may face an even more severe burden of LOC in the future.

Frontier analysis indicates that the largest effective gaps were observed in countries such as Pakistan and Palau. In Palau, over 70% of young people are current tobacco users, increasing their risk of developing LOC through DNA damage (47). Additionally, a survey of smokeless tobacco vendors in Pakistan found that 76% violated policies prohibiting tobacco sales to minors, particularly in rural areas (48). These findings underscore the need for differentiated targeted LOC prevention and tobacco control strategies tailored to the specific developmental stage and healthcare system characteristics of each country. Particular attention should be given to middle- and low-SDI countries with pronounced inequalities, employing measures such as enacting legislation, raising awareness about smoking cessation, and strengthening the enforcement of tobacco control policies to mitigate the negative impact of LOC.

Our study has several limitations. First, while GBD 2021 covers 204 countries and territories, differences in data availability and quality across regions may affect the accuracy of the analysis. Particularly in low SDI regions, due to insufficient healthcare resources and limitations in data collection, the mortality rate of LOC attributed to tobacco use may be underestimated. Second, the potential confounding effects of other factors, such as alcohol consumption, can influence LOC mortality rates. Finally, variations in data collection methods, techniques, and tools exist across different countries and regions. This heterogeneity in cross-national evidence can be confusing, making it difficult to distinguish between high- and low-quality data, which may subsequently impact the analysis of health inequalities.

## 5 Conclusions

This study provides a comprehensive and in-depth analysis of the slightly declining trend in the burden of LOC attributable to tobacco. Population growth is the primary driver of the increasing burden. As SDI levels rise, the burden of LOC tends to increase, with significant health inequalities observed between regions. This underscores the necessity for global health policies to focus on reducing the burden of LOC in high-income countries and regions, while also addressing the gradually increasing trend of LOC burden in low SDI areas. Projected estimates suggest a slight decrease in DALYs and deaths attributable to tobacco for LOC by 2050. This indicates that continued efforts in formulating and implementing tobacco control policies remain essential to reduce the global burden of LOC.

## Data Availability

The original contributions presented in the study are included in the article/**Supplementary Material**. Further inquiries can be directed to the corresponding author.

## Supplementary material

Supplementary Figure 1 Global distribution map of EAPC for age-standardized rates of lip and oral cancer caused by tobacco between 1990 and 2021. age-standardized mortality rate **(A)** and age-standardized DALY rate **(B)**.

Supplementary Figure 2 Age-Period-Cohort analysis of lip and oral cancer DALY attributable to tobacco from 1990 to 2021. **(A)** Longitudinal age curve of lip and oral cancer DALY; **(B)** Cross-sectional age curve of lip and oral cancer DALY; **(C)** Comparison of longitudinal to cross-sectional rate ratios; **(D)** Fitted temporal trend of lip and oral cancer DALY; **(E)** Period rate ratios over time; **(F)** Cohort rate ratios by birth cohort; **(G)** Local drifts of lip and oral cancer DALY by age; **(H)** Age deviation from the fitted model; **(I)** Period deviations from the fitted model; **(J)** Cohort deviation from the fitted model; **(K)** Fitted cohort pattern of lip and oral cancer DALY.

Supplementary Figure 3 Between 1990 and 2021, the determinants of aging, population growth, and epidemiological changes in lip and oral cancer caused by tobacco in 21 GBD regions. Black dots indicate the net total change in disease burden. **(A)** Number of deaths; **(B)** Disability-adjusted life years.

## References

[1] Screening PDQ Prevention Editorial B . Oral Cavity, Oropharyngeal, Hypopharyngeal, and Laryngeal Cancers Prevention (PDQ^®^): Health Professional Version. PDQ Cancer Information Summaries. Bethesda (MD: National Cancer Institute (US (2002). 26389416

[2] Zini A Czerninski R Sgan-Cohen HD . Oral cancer over four decades: epidemiology, trends, histology, and survival by anatomical sites. J Oral Pathol Med. (2010) 39:299–305. doi: 10.1111/j.1600-0714.2009.00845.x, PMID: 20040019

[3] Akashanand Zahiruddin QS Jena D Ballal S Kumar S Bhat M . Burden of oral cancer and associated risk factors at national and state levels: A systematic analysis from the global burden of disease in India, 1990-2021. Oral Oncol. (2024) 159:107063. doi: 10.1016/j.oraloncology.2024.107063, PMID: 39357385

[4] . doi: 10.3322/caac.21660, PMID: 33538338

[5] Kumar M Nanavati R Modi TG Dobariya C . Oral cancer: Etiology and risk factors: A review. J Cancer Res Ther. (2016) 12:458–63. doi: 10.4103/0973-1482.186696, PMID: 27461593

[6] Gangane NM Ghongade PV Patil BU Atram M . Oral cavity cancer incidence and survival trends: A population-based study. J Cancer Res Ther. (2024) 20:1446–52. doi: 10.4103/jcrt.jcrt_2720_22, PMID: 38261454

[7] Irani S . Metastasis to head and neck area: a 16-year retrospective study. Am J otolaryngology. (2011) 32:24–7. doi: 10.1016/j.amjoto.2009.09.006, PMID: 20031269

[8] Ye L Jiang Y Liu W Tao H . Correlation between periodontal disease and oral cancer risk: A meta-analysis. J Cancer Res Ther. (2016) 12:C237–c40. doi: 10.4103/0973-1482.200746, PMID: 28230025

[9] Gallagher KP Vargas PA Santos-Silva AR . The use of E-cigarettes as a risk factor for oral potentially Malignant disorders and oral cancer: a rapid review of clinical evidence. Med oral patologia Oral y cirugia bucal. (2024) 29:e18–26. doi: 10.4317/medoral.26042, PMID: 37992145 PMC10765326

[10] Rodríguez-Molinero J Migueláñez-Medrán BDC Puente-Gutiérrez C Delgado-Somolinos E Martín Carreras-Presas C Fernández-Farhall J . Association between oral cancer and diet: an update. Nutrients. (2021) 13:1299. doi: 10.3390/nu13041299, PMID: 33920788 PMC8071138

[11] Yu ZW Zheng M Fan HY Liang XH Tang YL . Ultraviolet (UV) radiation: a double-edged sword in cancer development and therapy. Mol biomedicine. (2024) 5:49. doi: 10.1186/s43556-024-00209-8, PMID: 39417901 PMC11486887

[12] Warnakulasuriya S Chen THH . Areca nut and oral cancer: evidence from studies conducted in humans. J Dental Res. (2022) 101:1139–46. doi: 10.1177/00220345221092751, PMID: 35459408 PMC9397398

[13] Tan Y Wang Z Xu M Li B Huang Z Qin S . Oral squamous cell carcinomas: state of the field and emerging directions. Int J Oral science. (2023) 15:44. doi: 10.1038/s41368-023-00249-w, PMID: 37736748 PMC10517027

[14] Winn DM . Tobacco use and oral disease. J Dental education. (2001) 65:306–12. doi: 10.1002/j.0022-0337.2001.65.4.tb03400.x 11336115

[15] Chaturvedi P Singh A Chien CY Warnakulasuriya S . Tobacco related oral cancer. BMJ (Clinical Res ed). (2019) 365:l2142. doi: 10.1136/bmj.l2142, PMID: 31167798

[16] Jerjes W Upile T Radhi H Petrie A Abiola J Adams A . The effect of tobacco and alcohol and their reduction/cessation on mortality in oral cancer patients: short communication. Head Neck Oncol. (2012) 4:6. doi: 10.1186/1758-3284-4-6, PMID: 22409767 PMC3329636

[17] Cunha ARD Compton K Xu R Mishra R Drangsholt MT Antunes JLF . The global, regional, and national burden of adult lip, oral, and pharyngeal cancer in 204 countries and territories: A systematic analysis for the global burden of disease study 2019. JAMA Oncol. (2023) 9:1401–16. doi: 10.1001/jamaoncol.2023.2960, PMID: 37676656 PMC10485745

[18] Yu Z Wu Y Cao Y Cheng P . Epidemiological trends and age-period-cohort effects on lip and oral cavity cancer burden across the BRICS from 1992 to 2021. Front Oncol. (2025) 15:1539417. doi: 10.3389/fonc.2025.1539417, PMID: 40034600 PMC11873103

[19] Yu Z Ma X Xiao H Chen Y Wu Y He J . Disease burden and attributable risk factors of lip and oral cavity cancer in China from 1990 to 2021 and its prediction to 2031. Front Public Health. (2024) 12:1419428. doi: 10.3389/fpubh.2024.1419428, PMID: 39310910 PMC11413874

[20] GBD 2021 Diseases and Injuries Collaborators . Global incidence, prevalence, years lived with disability (YLDs), disability-adjusted life-years (DALYs), and healthy life expectancy (HALE) for 371 diseases and injuries in 204 countries and territories and 811 subnational locations, 1990-2021: a systematic analysis for the Global Burden of Disease Study 2021. Lancet (London England). (2024) 403:2133–61. doi: 10.1016/S0140-6736(24)00757-8, PMID: 38642570 PMC11122111

[21] Zhao Y Peng X Zhong Z Pan W Zheng J Tian X . Epidemiological and demographic analysis of liver cancer attributable to modifiable risk factors from 1990 to 2021. Sci Rep. (2025) 15:19217. doi: 10.1038/s41598-025-02031-w, PMID: 40451844 PMC12127442

[22] Knoll M Furkel J Debus J Abdollahi A Karch A Stock C . An R package for an integrated evaluation of statistical approaches to cancer incidence projection. BMC Med Res methodology. (2020) 20:257. doi: 10.1186/s12874-020-01133-5, PMID: 33059585 PMC7559591

[23] Shi L Kuang Z Tu J Wang T Liu T Liu J . Global, regional, and national burden of laryngeal cancer attributable to smoking, 1990-2021, and projections to 2036: a systematic analysis of the Global Burden of Disease study 2021. Front Public Health. (2025) 13:1583045. doi: 10.3389/fpubh.2025.1583045, PMID: 40416653 PMC12098609

[24] Bai Z Han J An J Wang H Du X Yang Z . The global, regional, and national patterns of change in the burden of congenital birth defects, 1990-2021: an analysis of the global burden of disease study 2021 and forecast to 2040. EClinicalMedicine. (2024) 77:102873. doi: 10.1016/j.eclinm.2024.102873, PMID: 39416384 PMC11474384

[25] Koolman X van Doorslaer E . On the interpretation of a concentration index of inequality. Health Econ. (2004) 13:649–56. doi: 10.1002/hec.884, PMID: 15259044

[26] Available online at: www.who.int/publications/i/item/9789241548632 (Accessed June 19, 2025).

[27] Shrestha AD Vedsted P Kallestrup P Neupane D . Prevalence and incidence of oral cancer in low- and middle-income countries: A scoping review. Eur J Cancer Care. (2020) 29:7:e1320. doi: 10.1111/ecc.13207, PMID: 31820851

[28] Miranda-Filho A Bray F . Global patterns and trends in cancers of the lip, tongue and mouth. Oral Oncol. (2020) 102:104551. doi: 10.1016/j.oraloncology.2019.104551, PMID: 31986342

[29] Palipudi K Rizwan SA Sinha DN Andes LJ Amarchand R Krishnan A . Prevalence and sociodemographic determinants of tobacco use in four countries of the World Health Organization: South-East Asia region: findings from the Global Adult Tobacco Survey. Indian J cancer. (2014) 51 Suppl 1:S24–32. doi: 10.4103/0019-509X.147446, PMID: 25526244

[30] Cao LM Zhang B Luo HY Zhou K Yu YF Li ZZ . Areca nut production, imports, and their impact on oral cancer incidence. J Dental Res. (2025) 9:220345251344895. doi: 10.1177/00220345251344895, PMID: 40631888

[31] Kharbanda OP Ivaturi A Priya H Dorji G Gupta S . Digital possibilities in the prevention and early detection of oral cancer in the WHO South-East Asia Region. WHO South-East Asia J Public Health. (2019) 8:95–100. doi: 10.4103/2224-3151.264853, PMID: 31441444

[32] Gupta AK Kanaan M Siddiqi K Sinha DN Mehrotra R . Oral cancer risk assessment for different types of smokeless tobacco products sold worldwide: A review of reviews and meta-analyses. Cancer Prev Res (Philadelphia Pa). (2022) 15:733–46. doi: 10.1158/1940-6207.CAPR-21-0567, PMID: 36095092

[33] Bilano V Gilmour S Moffiet T d’Espaignet ET Stevens GA Commar A . Global trends and projections for tobacco use, 1990-2025: an analysis of smoking indicators from the WHO Comprehensive Information Systems for Tobacco Control. Lancet (London England). (2015) 385:966–76. doi: 10.1016/S0140-6736(15)60264-1, PMID: 25784347

[34] Jansen L Buttmann-Schweiger N Listl S Ressing M Holleczek B Katalinic A . Differences in incidence and survival of oral cavity and pharyngeal cancers between Germany and the United States depend on the HPV-association of the cancer site. Oral Oncol. (2018) 76:8–15. doi: 10.1016/j.oraloncology.2017.11.015, PMID: 29290288

[35] Scharbrodt R Habig S Kalab M Baumann E Felgendreff L Dempfle A . Knowledge level of diagnostic procedures and risk factors for oral cancer among oral healthcare providers in Germany. BMC Oral Health. (2025) 25:681. doi: 10.1186/s12903-025-06048-5, PMID: 40317003 PMC12048965

[36] Flor LS Reitsma MB Gupta V Ng M Gakidou E . The effects of tobacco control policies on global smoking prevalence. Nat Med. (2021) 27:239–43. doi: 10.1038/s41591-020-01210-8, PMID: 33479500 PMC7884287

[37] Bozhar H McKee M Spadea T Veerus P Heinävaara S Anttila A . Socio-economic inequality of utilization of cancer testing in Europe: A cross-sectional study. Prev Med Rep. (2022) 26:101733. doi: 10.1016/j.pmedr.2022.101733, PMID: 35198362 PMC8850331

[38] Cunha ARD Prass TS Hugo FN . Mortality from oral and oropharyngeal cancer in Brazil: impact of the National Oral Health Policy. Cadernos saude publica. (2019) 35:e00014319. doi: 10.1590/0102-311x00014319, PMID: 31800779

[39] Available online at: https://globalactiontoendsmoking.org/research/tobacco-around-the-world/Pakistan/(Accessed June 19, 2025).

[40] Hassan S Sifat A Munib M Saeed S Nisa WU Durrani SH . Cytomorphological changes of oral mucosal cells among smokeless tobacco users in low and middle-income country settings: new findings from Pakistan. BMC Oral Health. (2024) 24:1541. doi: 10.1186/s12903-024-05220-7, PMID: 39710646 PMC11665104

[41] Khan MF Hayhoe RP Kabir R . Exploring the risk factors for oral cancer in Pakistan: A systematic literature review. Dentistry J. (2024) 12:25. doi: 10.3390/dj12020025, PMID: 38392229 PMC10887545

[42] Santos T Hicks WL Jr. Magner WJ Al Afif A Kirkwood KL . Metabolic and aging influence on anticancer immunity in oral cancer. J Dental Res. (2024) 103:953–61. doi: 10.1177/00220345241264728, PMID: 39185914

[43] Patel J Wallace J Doshi M Gadanya M Ben Yahya I Roseman J . Oral health for healthy ageing. Lancet Healthy longevity. (2021) 2:e521–e7. doi: 10.1016/S2666-7568(21)00142-2, PMID: 36098001

[44] Chan KH Xiao D Zhou M Peto R Chen Z . Tobacco control in China. Lancet Public Health. (2023) 8:e1006–e15. doi: 10.1016/S2468-2667(23)00242-6, PMID: 38000880

[45] Chugh A Arora M Jain N Vidyasagaran A Readshaw A Sheikh A . The global impact of tobacco control policies on smokeless tobacco use: a systematic review. Lancet Global Health. (2023) 11:e953–e68. doi: 10.1016/S2214-109X(23)00205-X, PMID: 37202029

[46] Nyman AL Weaver SR Popova L Pechacek TF Huang J Ashley DL . Awareness and use of heated tobacco products among US adults, 2016-2017. Tobacco control. (2018) 27:s55–61. doi: 10.1136/tobaccocontrol-2018-054323, PMID: 30158204 PMC6218939

[47] Chiang C Singeo ST Jr. Yatsuya H Honjo K Mita T Ikerdeu E . Profile of non-communicable disease risk factors among young people in Palau. J Epidemiol. (2015) 25:392–7. doi: 10.2188/jea.JE20140156, PMID: 25787240 PMC4411239

[48] Ahmad F Khan Z Siddiqi K Khan MN Kibria Z Forberger S . Awareness, perceptions of and compliance with tobacco control policies among naswar vendors in Khyber Pakhtunkhwa Pakistan. Tobacco control. (2022) 31:e111–e7. doi: 10.1136/tobaccocontrol-2020-056377, PMID: 34226260

