# Supplementary figures and images for: Study on the disease burden of lip and oral cancer attributable to tobacco use: based on the 2021 Global Burden of Disease study
Source: Front Oncol. 2025 Nov 7;15:1690271. doi: 10.3389/fonc.2025.1690271 (PMC12634366; doi:10.3389/fonc.2025.1690271)

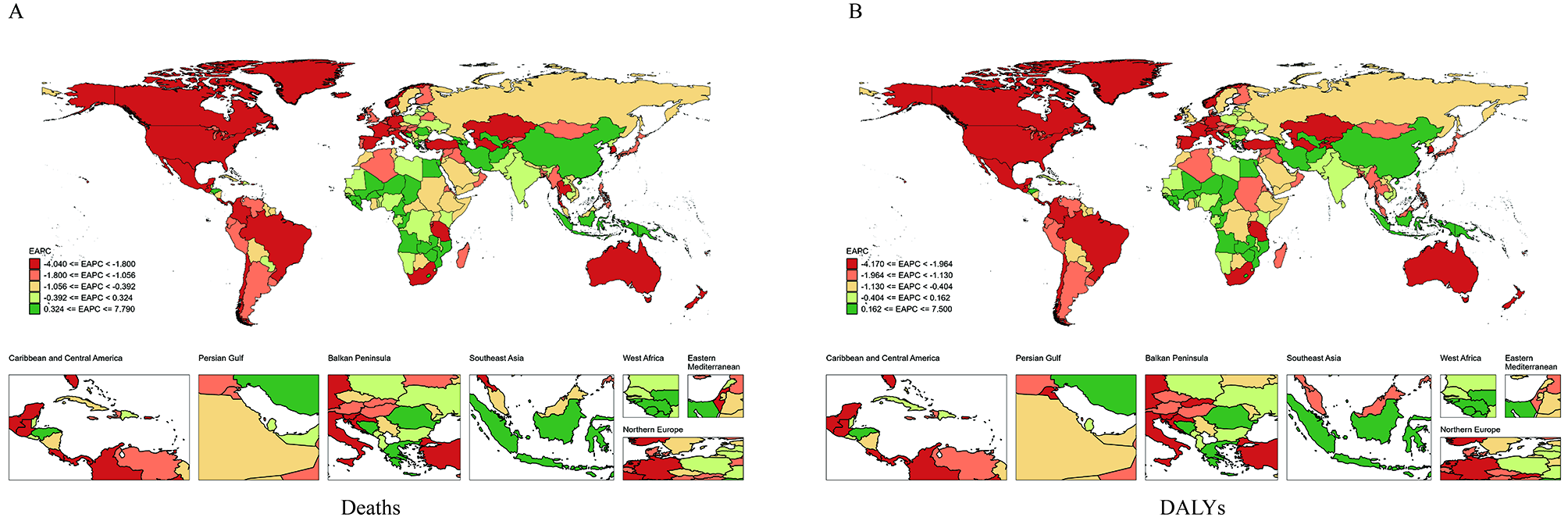

Supplement: Supplementary Figure 1 — Global distribution map of EAPC for age-standardized rates of lip and oral cancer caused by tobacco between 1990 and 2021. age-standardized mortality rate (A) and age-standardized DALY rate (B). [file Image1.tif]

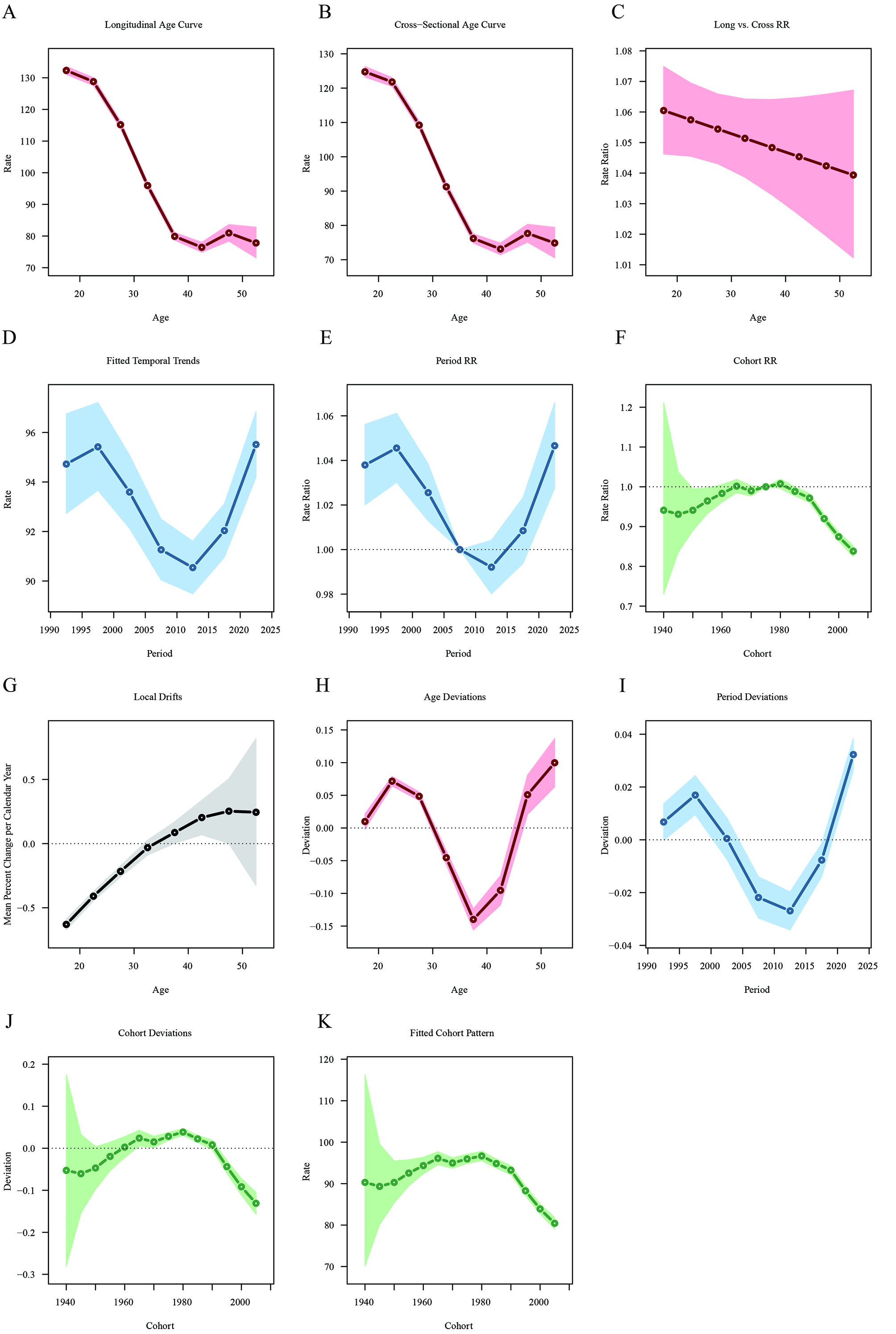

Supplement: Supplementary Figure 2 — Age-Period-Cohort analysis of lip and oral cancer DALY attributable to tobacco from 1990 to 2021. (A) Longitudinal age curve of lip and oral cancer DALY; (B) Cross-sectional age curve of lip and oral cancer DALY; (C) Comparison of longitudinal to cross-sectional rate ratios; (D) Fitted temporal trend of lip and oral cancer DALY; (E) Period rate ratios over time; (F) Cohort rate ratios by birth cohort; (G) Local drifts of lip and oral cancer DALY by age; (H) Age deviation from the fitted model; (I) Period deviations from the fitted model; (J) Cohort deviation from the fitted model; (K) Fitted cohort pattern of lip and oral cancer DALY. [file Image2.tif]

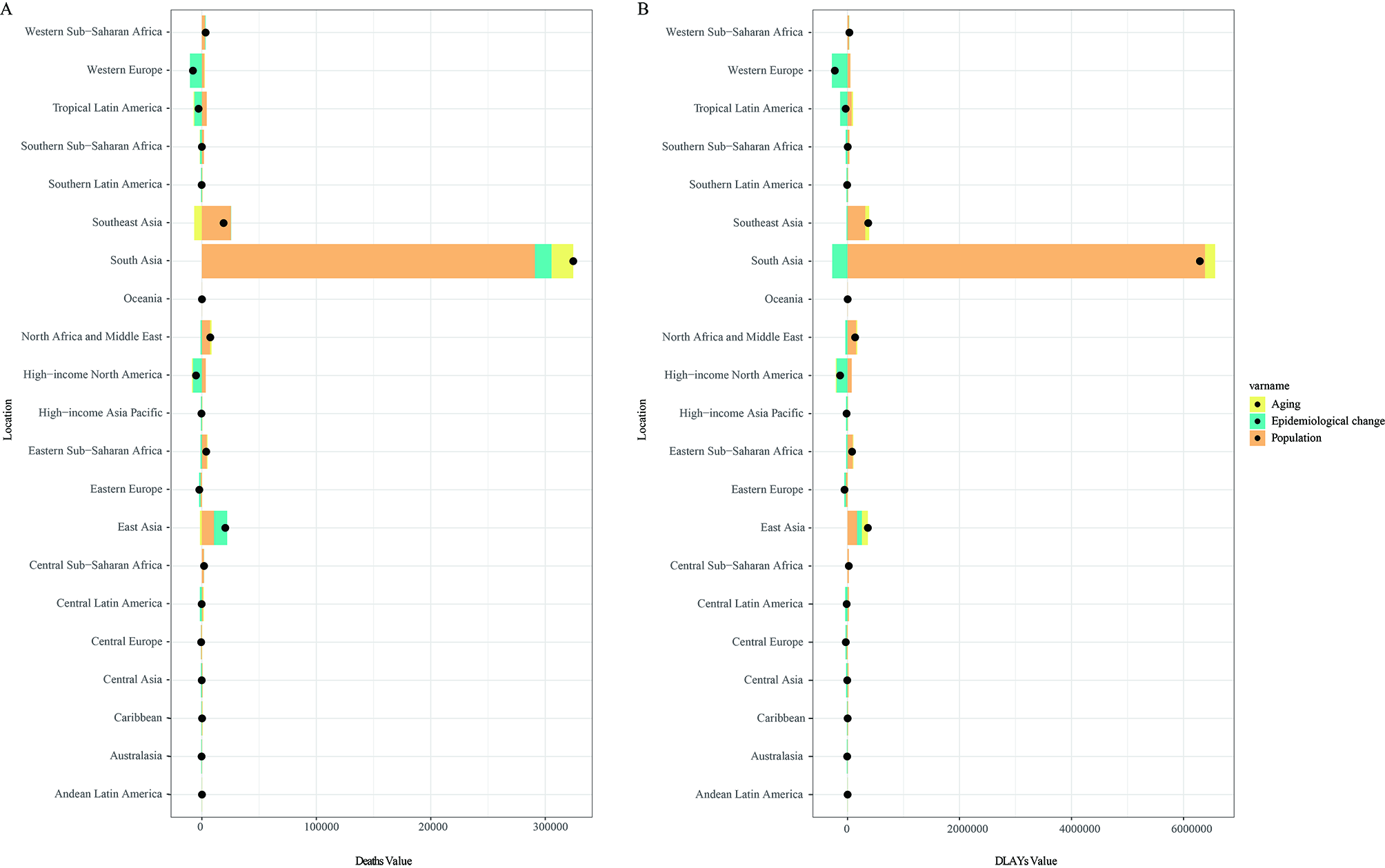

Supplement: Supplementary Figure 3 — Between 1990 and 2021, the determinants of aging, population growth, and epidemiological changes in lip and oral cancer caused by tobacco in 21 GBD regions. Black dots indicate the net total change in disease burden. (A) Number of deaths; (B) Disability-adjusted life years. [file Image3.tif]
