# Supplementary material for: Study on the disease burden of lip and oral cancer attributable to tobacco use: based on the 2021 Global Burden of Disease study
Source: Front Oncol. 2025 Nov 7;15:1690271. doi: 10.3389/fonc.2025.1690271 (PMC12634366; doi:10.3389/fonc.2025.1690271)
Supplement: Supplementary file 4 [file Table1.docx]

Supplementary Table 1 The global disease burden of Lip and oral cavity cancer caused by tobacco in 204 countries and territories.

| location | 1990 |  | 2021 |  | EAPC_CI |
| --- | --- | --- | --- | --- | --- |
|  | Number | ASR | Number | ASR |  |
| **Deaths** |  |  |  |  |  |
| Afghanistan | 6(3-14) | 0.09(0.054-0.192) | 11(7-20) | 0.106(0.063-0.178) | 0.55 (0.42 to 0.69) |
| Albania | 14(9-20) | 0.66(0.435-0.956) | 26(16-39) | 0.609(0.372-0.927) | 0.11 (-0.04 to 0.25) |
| Algeria | 41(27-56) | 0.386(0.257-0.528) | 84(52-124) | 0.266(0.168-0.387) | -1.12 (-1.25 to -0.99) |
| American Samoa | 0(0-0) | 0.106(0.068-0.147) | 0(0-0) | 0.127(0.088-0.176) | 1.41 (0.94 to 1.87) |
| Andorra | 0(0-0) | 0.525(0.322-0.829) | 0(0-1) | 0.263(0.14-0.42) | -2.07 (-2.22 to -1.91) |
| Angola | 12(7-20) | 0.31(0.19-0.487) | 40(25-59) | 0.336(0.208-0.487) | 0.34 (0.17 to 0.51) |
| Antigua and Barbuda | 0(0-0) | 0.372(0.233-0.542) | 0(0-0) | 0.275(0.175-0.411) | -0.96 (-1.29 to -0.63) |
| Argenti | 193(133-255) | 0.589(0.405-0.782) | 191(127-264) | 0.35(0.233-0.483) | -1.35 (-1.68 to -1.01) |
| Armenia | 13(9-16) | 0.434(0.321-0.568) | 21(16-27) | 0.48(0.351-0.616) | 0.6 (0.29 to 0.92) |
| Australia | 113(77-151) | 0.583(0.397-0.773) | 107(68-155) | 0.246(0.159-0.355) | -2.78 (-3.06 to -2.49) |
| Austria | 85(58-111) | 0.804(0.558-1.049) | 92(61-123) | 0.553(0.37-0.74) | -1.2 (-1.35 to -1.06) |
| Azerbaijan | 12(7-18) | 0.223(0.135-0.347) | 24(13-39) | 0.222(0.124-0.352) | 0.41 (0.18 to 0.63) |
| Bahamas | 1(1-1) | 0.548(0.334-0.801) | 2(1-3) | 0.43(0.256-0.65) | -0.49 (-0.69 to -0.29) |
| Bahrain | 1(1-1) | 0.595(0.409-0.834) | 3(2-5) | 0.402(0.231-0.596) | -1.39 (-1.56 to -1.22) |
| Bangladesh | 1912(1280-2547) | 4.123(2.752-5.492) | 3994(2571-5817) | 2.965(1.948-4.289) | -1.1 (-1.18 to -1.03) |
| Barbados | 1(1-2) | 0.436(0.279-0.627) | 1(1-2) | 0.269(0.158-0.413) | -1.67 (-1.86 to -1.48) |
| Belarus | 241(176-305) | 1.832(1.338-2.308) | 217(147-295) | 1.396(0.946-1.897) | -1.13 (-1.38 to -0.88) |
| Belgium | 120(85-155) | 0.837(0.593-1.076) | 114(74-154) | 0.539(0.356-0.723) | -1.57 (-1.8 to -1.34) |
| Belize | 0(0-0) | 0.229(0.158-0.31) | 1(0-1) | 0.187(0.124-0.266) | -0.33 (-0.73 to 0.08) |
| Benin | 3(2-4) | 0.14(0.096-0.205) | 7(5-11) | 0.158(0.098-0.246) | 0.19 (0.02 to 0.37) |
| Bermuda | 0(0-1) | 0.771(0.496-1.126) | 1(0-1) | 0.381(0.241-0.565) | -1.79 (-2.09 to -1.48) |
| Bhutan | 6(4-9) | 2.686(1.781-3.714) | 14(9-19) | 2.281(1.582-3.161) | -0.63 (-0.74 to -0.52) |
| Bolivia (Pluritiol State of) | 5(3-9) | 0.174(0.105-0.291) | 11(7-18) | 0.13(0.079-0.206) | -0.57 (-0.79 to -0.35) |
| Bosnia and Herzegovi | 30(21-40) | 0.684(0.483-0.903) | 45(30-63) | 0.752(0.505-1.041) | 0.68 (0.47 to 0.89) |
| Botswa | 6(4-9) | 1.146(0.787-1.641) | 14(9-20) | 0.974(0.654-1.336) | -0.62 (-0.8 to -0.45) |
| Brazil | 951(691-1209) | 1.066(0.772-1.367) | 1316(896-1784) | 0.517(0.351-0.702) | -2.55 (-2.7 to -2.4) |
| Brunei Darussalam | 1(1-2) | 1.167(0.802-1.624) | 2(1-3) | 0.621(0.415-0.856) | -1.63 (-1.93 to -1.34) |
| Bulgaria | 85(61-109) | 0.703(0.505-0.901) | 107(72-147) | 0.897(0.604-1.223) | 0.15 (-0.19 to 0.49) |
| Burki Faso | 7(5-11) | 0.168(0.107-0.253) | 19(12-29) | 0.211(0.135-0.321) | 0.6 (0.46 to 0.74) |
| Burundi | 17(11-23) | 0.722(0.481-1) | 20(12-30) | 0.383(0.24-0.569) | -2.25 (-2.34 to -2.15) |
| Cabo Verde | 0(0-0) | 0.026(0.018-0.036) | 2(2-4) | 0.529(0.339-0.783) | 7.79 (5.22 to 10.42) |
| Cambodia | 60(43-83) | 1.495(1.094-2.03) | 179(124-242) | 1.623(1.146-2.158) | 0.2 (0.15 to 0.25) |
| Cameroon | 8(6-12) | 0.197(0.139-0.286) | 27(17-39) | 0.248(0.162-0.361) | 0.67 (0.5 to 0.85) |
| Cada | 309(222-403) | 0.963(0.692-1.255) | 263(175-365) | 0.364(0.246-0.502) | -3.01 (-3.19 to -2.83) |
| Central African Republic | 3(2-5) | 0.273(0.175-0.44) | 5(3-8) | 0.223(0.144-0.344) | -0.78 (-0.91 to -0.66) |
| Chad | 3(2-5) | 0.125(0.084-0.183) | 10(6-14) | 0.181(0.117-0.266) | 1.07 (0.86 to 1.29) |
| Chile | 27(18-36) | 0.261(0.173-0.353) | 30(20-41) | 0.117(0.077-0.163) | -2.51 (-2.67 to -2.35) |
| Chi | 3879(2844-4960) | 0.478(0.35-0.611) | 10388(7072-14289) | 0.487(0.332-0.667) | 0.33 (0.11 to 0.56) |
| Colombia | 53(37-73) | 0.317(0.216-0.441) | 60(40-87) | 0.108(0.071-0.157) | -4.04 (-4.26 to -3.82) |
| Comoros | 2(1-3) | 1.027(0.684-1.397) | 4(3-6) | 0.837(0.558-1.216) | -0.98 (-1.1 to -0.87) |
| Congo | 3(2-5) | 0.298(0.186-0.464) | 8(5-12) | 0.326(0.222-0.462) | 0.37 (0.23 to 0.51) |
| Cook Islands | 0(0-0) | 0.338(0.211-0.478) | 0(0-0) | 0.286(0.175-0.436) | -0.44 (-0.48 to -0.39) |
| Costa Rica | 7(5-9) | 0.4(0.266-0.552) | 10(6-15) | 0.187(0.116-0.265) | -2.65 (-2.8 to -2.5) |
| Croatia | 114(82-147) | 1.806(1.286-2.338) | 80(54-109) | 0.964(0.66-1.314) | -1.8 (-1.91 to -1.7) |
| Cuba | 108(76-140) | 1.063(0.752-1.385) | 174(115-233) | 0.879(0.588-1.18) | -0.44 (-0.56 to -0.33) |
| Cyprus | 4(3-6) | 0.544(0.342-0.763) | 6(4-9) | 0.322(0.204-0.451) | -1.61 (-1.71 to -1.52) |
| Czechia | 153(105-202) | 1.15(0.793-1.517) | 159(107-220) | 0.82(0.559-1.136) | -1.04 (-1.13 to -0.95) |
| C么te d'Ivoire | 10(6-14) | 0.241(0.159-0.356) | 28(16-44) | 0.249(0.15-0.387) | -0.09 (-0.25 to 0.08) |
| Democratic People's Republic of Korea | 60(39-85) | 0.354(0.234-0.499) | 122(74-182) | 0.358(0.218-0.532) | 0.08 (0.02 to 0.15) |
| Democratic Republic of the Congo | 34(22-48) | 0.223(0.144-0.317) | 71(45-105) | 0.201(0.125-0.306) | -0.38 (-0.48 to -0.28) |
| Denmark | 51(37-63) | 0.667(0.494-0.824) | 61(40-82) | 0.528(0.349-0.701) | -1.57 (-2.15 to -0.98) |
| Djibouti | 1(1-2) | 0.778(0.469-1.217) | 4(2-6) | 0.689(0.417-1.03) | -0.4 (-0.49 to -0.3) |
| Dominica | 0(0-0) | 0.443(0.275-0.642) | 0(0-0) | 0.336(0.198-0.508) | -0.81 (-0.94 to -0.68) |
| Dominican Republic | 26(16-38) | 0.771(0.496-1.103) | 64(38-99) | 0.65(0.383-1.01) | -0.39 (-0.65 to -0.13) |
| Ecuador | 8(5-11) | 0.161(0.11-0.222) | 14(9-22) | 0.09(0.055-0.136) | -1.21 (-1.45 to -0.98) |
| Egypt | 26(18-33) | 0.105(0.074-0.14) | 95(66-130) | 0.167(0.113-0.227) | 2.09 (1.62 to 2.57) |
| El Salvador | 4(2-5) | 0.122(0.078-0.179) | 7(4-11) | 0.116(0.073-0.174) | -0.11 (-0.21 to 0) |
| Equatorial Guinea | 0(0-1) | 0.224(0.143-0.352) | 1(1-2) | 0.265(0.16-0.41) | 0.58 (0.51 to 0.65) |
| Eritrea | 5(3-8) | 0.354(0.202-0.574) | 9(5-13) | 0.276(0.164-0.416) | -1.2 (-1.37 to -1.03) |
| Estonia | 16(12-21) | 0.806(0.567-1.045) | 17(12-24) | 0.745(0.506-1.009) | -0.81 (-1.13 to -0.5) |
| Eswatini | 1(1-2) | 0.427(0.289-0.642) | 2(1-3) | 0.367(0.225-0.578) | -0.41 (-0.67 to -0.15) |
| Ethiopia | 40(24-70) | 0.22(0.136-0.374) | 69(47-97) | 0.178(0.122-0.251) | -0.64 (-0.77 to -0.52) |
| Fiji | 2(1-2) | 0.453(0.3-0.636) | 2(2-4) | 0.303(0.202-0.434) | -1.26 (-1.39 to -1.13) |
| Finland | 26(17-35) | 0.391(0.264-0.524) | 27(17-37) | 0.245(0.158-0.336) | -1.34 (-1.44 to -1.25) |
| France | 1135(785-1496) | 1.499(1.044-1.958) | 614(389-844) | 0.494(0.318-0.668) | -3.53 (-3.69 to -3.37) |
| Gabon | 1(1-2) | 0.23(0.148-0.34) | 3(2-4) | 0.248(0.154-0.359) | 0.16 (0.07 to 0.25) |
| Gambia | 1(0-1) | 0.17(0.113-0.241) | 2(1-2) | 0.161(0.102-0.239) | -0.45 (-0.6 to -0.3) |
| Georgia | 41(28-54) | 0.626(0.428-0.838) | 52(37-67) | 0.892(0.645-1.153) | 2.01 (1.35 to 2.68) |
| Germany | 1107(772-1421) | 0.948(0.666-1.212) | 872(561-1200) | 0.501(0.324-0.679) | -1.97 (-2.11 to -1.82) |
| Gha | 2(1-2) | 0.028(0.019-0.042) | 4(3-6) | 0.026(0.016-0.039) | -0.63 (-0.95 to -0.31) |
| Greece | 70(52-87) | 0.468(0.347-0.581) | 111(77-148) | 0.517(0.365-0.67) | 0.36 (0.22 to 0.49) |
| Greenland | 1(1-1) | 2.712(1.986-3.499) | 1(1-1) | 1.157(0.781-1.605) | -2.83 (-2.93 to -2.72) |
| Greda | 0(0-1) | 0.526(0.342-0.77) | 0(0-1) | 0.312(0.194-0.462) | -1.51 (-1.89 to -1.14) |
| Guam | 0(0-0) | 0.2(0.14-0.275) | 1(0-1) | 0.36(0.248-0.485) | 2.86 (2.61 to 3.12) |
| Guatemala | 5(3-7) | 0.177(0.116-0.254) | 9(6-13) | 0.087(0.057-0.125) | -2.84 (-3.08 to -2.6) |
| Guinea | 12(9-17) | 0.385(0.265-0.541) | 24(16-35) | 0.44(0.292-0.637) | 0.47 (0.38 to 0.55) |
| Guinea-Bissau | 0(0-1) | 0.104(0.063-0.16) | 1(1-2) | 0.17(0.111-0.252) | 1.72 (1.66 to 1.78) |
| Guya | 1(1-2) | 0.277(0.182-0.398) | 1(1-2) | 0.196(0.115-0.304) | -0.51 (-0.69 to -0.33) |
| Haiti | 9(5-17) | 0.277(0.161-0.501) | 12(7-20) | 0.164(0.092-0.276) | -1.73 (-1.85 to -1.61) |
| Honduras | 4(2-5) | 0.196(0.124-0.274) | 13(8-20) | 0.211(0.133-0.337) | 0.34 (0.26 to 0.42) |
| Hungary | 303(215-398) | 2.172(1.545-2.843) | 251(167-341) | 1.482(0.995-2.008) | -1.58 (-1.99 to -1.16) |
| Iceland | 2(1-2) | 0.631(0.443-0.808) | 2(1-3) | 0.35(0.23-0.482) | -2.09 (-2.21 to -1.96) |
| India | 13881(11380-16750) | 2.953(2.419-3.531) | 34443(27798-41433) | 2.93(2.377-3.495) | -0.17 (-0.26 to -0.08) |
| Indonesia | 533(385-685) | 0.624(0.453-0.798) | 1494(996-2010) | 0.733(0.494-0.989) | 0.4 (0.29 to 0.51) |
| Iran (Islamic Republic of) | 20(14-28) | 0.083(0.056-0.112) | 67(45-87) | 0.088(0.06-0.117) | 0.57 (0.42 to 0.72) |
| Iraq | 27(17-38) | 0.338(0.218-0.481) | 63(37-91) | 0.268(0.16-0.384) | -1.06 (-1.17 to -0.95) |
| Ireland | 41(29-52) | 1.014(0.723-1.295) | 27(17-38) | 0.348(0.225-0.482) | -3.45 (-3.63 to -3.27) |
| Israel | 16(11-21) | 0.337(0.236-0.445) | 24(16-33) | 0.199(0.131-0.276) | -1.84 (-2.05 to -1.63) |
| Italy | 812(570-1032) | 0.948(0.669-1.202) | 556(364-760) | 0.412(0.274-0.555) | -2.67 (-2.85 to -2.49) |
| Jamaica | 5(4-8) | 0.313(0.213-0.431) | 7(4-11) | 0.221(0.13-0.343) | -1.27 (-1.7 to -0.84) |
| Japan | 669(505-825) | 0.393(0.297-0.486) | 1157(795-1578) | 0.324(0.23-0.43) | -1.12 (-1.52 to -0.72) |
| Jordan | 6(4-8) | 0.429(0.296-0.586) | 24(15-35) | 0.336(0.217-0.477) | -0.74 (-0.81 to -0.67) |
| Kazakhstan | 120(84-157) | 0.884(0.621-1.162) | 97(68-129) | 0.504(0.349-0.673) | -2.19 (-2.44 to -1.94) |
| Kenya | 41(25-61) | 0.521(0.313-0.76) | 118(82-160) | 0.526(0.363-0.707) | 0.02 (-0.05 to 0.09) |
| Kiribati | 1(0-1) | 1.771(1.2-2.426) | 2(1-2) | 1.998(1.263-2.764) | 0.43 (0.36 to 0.5) |
| Kuwait | 3(2-4) | 0.483(0.332-0.638) | 4(3-6) | 0.142(0.092-0.203) | -1.68 (-2.69 to -0.66) |
| Kyrgyzstan | 49(36-62) | 1.61(1.189-2.023) | 30(22-39) | 0.585(0.425-0.76) | -1.64 (-2.49 to -0.77) |
| Lao People's Democratic Republic | 21(14-32) | 1.112(0.773-1.631) | 40(28-55) | 0.995(0.7-1.344) | -0.34 (-0.41 to -0.26) |
| Latvia | 39(28-51) | 1.082(0.787-1.429) | 36(25-50) | 1.062(0.725-1.448) | -0.12 (-0.31 to 0.07) |
| Lebanon | 10(6-17) | 0.465(0.296-0.772) | 25(16-35) | 0.414(0.27-0.582) | -0.25 (-0.46 to -0.04) |
| Lesotho | 6(4-9) | 0.725(0.477-1.031) | 12(7-21) | 1.157(0.695-1.895) | 1.98 (1.78 to 2.17) |
| Liberia | 1(1-1) | 0.084(0.053-0.127) | 2(1-3) | 0.098(0.061-0.153) | 0.46 (0.35 to 0.57) |
| Libya | 6(4-10) | 0.335(0.197-0.522) | 17(11-25) | 0.307(0.197-0.456) | -0.1 (-0.18 to -0.02) |
| Lithuania | 39(27-51) | 0.866(0.592-1.122) | 52(36-71) | 1.044(0.719-1.411) | 0.15 (-0.07 to 0.37) |
| Luxembourg | 6(4-7) | 1.053(0.727-1.393) | 5(3-6) | 0.441(0.28-0.601) | -2.78 (-2.9 to -2.66) |
| Madagascar | 47(32-63) | 0.873(0.611-1.171) | 72(46-105) | 0.537(0.344-0.779) | -1.66 (-1.75 to -1.57) |
| Malawi | 14(10-19) | 0.378(0.267-0.516) | 29(19-39) | 0.401(0.267-0.537) | 0.09 (-0.02 to 0.19) |
| Malaysia | 93(68-120) | 1.051(0.762-1.357) | 228(169-297) | 0.847(0.627-1.11) | -1.03 (-1.2 to -0.86) |
| Maldives | 1(1-2) | 1.519(1.085-2.168) | 3(2-4) | 0.965(0.704-1.233) | -1.83 (-1.97 to -1.7) |
| Mali | 6(4-8) | 0.157(0.104-0.218) | 16(10-23) | 0.187(0.116-0.267) | 0.75 (0.64 to 0.86) |
| Malta | 3(2-3) | 0.597(0.417-0.792) | 3(2-4) | 0.342(0.223-0.475) | -1.85 (-1.99 to -1.72) |
| Marshall Islands | 0(0-0) | 0.291(0.182-0.47) | 0(0-0) | 0.378(0.212-0.616) | 1.09 (0.98 to 1.21) |
| Mauritania | 1(1-2) | 0.139(0.093-0.194) | 3(2-5) | 0.155(0.098-0.229) | 0.12 (0.05 to 0.19) |
| Mauritius | 8(6-10) | 1.246(0.972-1.502) | 17(13-21) | 0.928(0.72-1.138) | -0.46 (-0.74 to -0.17) |
| Mexico | 113(80-148) | 0.304(0.214-0.4) | 132(90-179) | 0.109(0.074-0.148) | -3.46 (-3.6 to -3.32) |
| Micronesia (Federated States of) | 0(0-0) | 0.455(0.295-0.682) | 0(0-1) | 0.5(0.332-0.754) | 0.41 (0.35 to 0.47) |
| Moco | 0(0-0) | 0.289(0.182-0.414) | 0(0-0) | 0.233(0.144-0.352) | -0.68 (-0.77 to -0.59) |
| Mongolia | 7(4-11) | 0.677(0.4-1.007) | 11(6-16) | 0.446(0.265-0.682) | -1.64 (-1.92 to -1.36) |
| Montenegro | 6(4-8) | 0.96(0.662-1.26) | 9(6-13) | 0.944(0.611-1.312) | -0.11 (-0.25 to 0.03) |
| Morocco | 20(13-29) | 0.142(0.089-0.204) | 40(24-60) | 0.112(0.067-0.169) | -0.85 (-1.01 to -0.68) |
| Mozambique | 16(11-22) | 0.293(0.211-0.399) | 32(21-45) | 0.306(0.208-0.416) | 0.44 (0.34 to 0.55) |
| Myanmar | 246(167-387) | 1.15(0.799-1.727) | 366(253-506) | 0.798(0.559-1.103) | -1.36 (-1.43 to -1.28) |
| mibia | 6(4-9) | 1.074(0.752-1.513) | 13(8-18) | 1.036(0.698-1.424) | -0.29 (-0.51 to -0.07) |
| uru | 0(0-0) | 0.478(0.275-0.808) | 0(0-0) | 0.468(0.255-0.803) | 0.01 (-0.07 to 0.1) |
| Nepal | 283(195-384) | 3.141(2.14-4.204) | 613(423-831) | 2.745(1.925-3.681) | -0.44 (-0.62 to -0.26) |
| Netherlands | 138(101-174) | 0.714(0.523-0.898) | 134(88-186) | 0.376(0.251-0.518) | -1.9 (-2.05 to -1.75) |
| New Zealand | 22(15-29) | 0.566(0.384-0.756) | 23(15-34) | 0.272(0.18-0.396) | -2.19 (-2.54 to -1.84) |
| Nicaragua | 2(1-3) | 0.126(0.077-0.181) | 4(3-7) | 0.093(0.058-0.14) | -0.69 (-0.83 to -0.55) |
| Niger | 2(1-3) | 0.087(0.055-0.128) | 8(5-14) | 0.11(0.066-0.176) | 0.72 (0.6 to 0.85) |
| Nigeria | 18(13-26) | 0.046(0.031-0.066) | 36(23-54) | 0.041(0.027-0.061) | -0.33 (-0.41 to -0.25) |
| Niue | 0(0-0) | 0.32(0.196-0.47) | 0(0-0) | 0.343(0.219-0.523) | 0.13 (0.09 to 0.16) |
| North Macedonia | 15(10-19) | 0.744(0.502-0.992) | 23(15-31) | 0.692(0.451-0.951) | -0.19 (-0.45 to 0.06) |
| Northern Maria Islands | 0(0-0) | 0.855(0.546-1.255) | 1(1-1) | 1.557(1.01-2.187) | 2.97 (2.49 to 3.45) |
| Norway | 41(29-52) | 0.626(0.455-0.797) | 26(18-35) | 0.259(0.179-0.349) | -3.27 (-3.55 to -3) |
| Oman | 2(1-3) | 0.262(0.151-0.418) | 3(2-5) | 0.156(0.094-0.235) | -1.24 (-1.48 to -1) |
| Pakistan | 3459(2577-4345) | 6.317(4.722-7.927) | 7285(5510-9657) | 6.103(4.649-8.076) | -0.37 (-0.56 to -0.18) |
| Palau | 1(0-1) | 5.262(3.665-7.199) | 1(1-1) | 4.571(3.166-6.254) | -0.41 (-0.45 to -0.38) |
| Palestine | 1(1-2) | 0.19(0.123-0.279) | 4(2-5) | 0.156(0.106-0.213) | -0.82 (-0.95 to -0.69) |
| Pama | 5(3-7) | 0.36(0.245-0.488) | 8(5-12) | 0.175(0.112-0.262) | -2.53 (-2.71 to -2.35) |
| Papua New Guinea | 5(3-7) | 0.244(0.13-0.369) | 17(10-26) | 0.281(0.16-0.425) | 0.52 (0.41 to 0.63) |
| Paraguay | 13(9-18) | 0.617(0.415-0.833) | 33(21-49) | 0.571(0.369-0.851) | -0.39 (-0.59 to -0.19) |
| Peru | 10(6-14) | 0.084(0.054-0.124) | 22(13-35) | 0.066(0.039-0.104) | -1.1 (-1.29 to -0.9) |
| Philippines | 244(166-317) | 0.91(0.616-1.17) | 485(354-633) | 0.623(0.455-0.806) | -1.34 (-1.5 to -1.18) |
| Poland | 473(356-587) | 1.1(0.827-1.364) | 686(479-896) | 1.03(0.724-1.34) | -0.15 (-0.26 to -0.05) |
| Portugal | 116(78-152) | 0.854(0.574-1.129) | 106(71-146) | 0.532(0.363-0.724) | -1.42 (-1.56 to -1.27) |
| Puerto Rico | 23(15-34) | 0.645(0.405-0.95) | 16(9-25) | 0.235(0.138-0.37) | -2.97 (-3.16 to -2.78) |
| Qatar | 0(0-0) | 0.197(0.119-0.301) | 2(1-3) | 0.164(0.082-0.263) | -0.29 (-0.85 to 0.27) |
| Republic of Korea | 126(92-162) | 0.43(0.309-0.555) | 253(162-353) | 0.27(0.173-0.377) | -2.15 (-2.5 to -1.8) |
| Republic of Moldova | 55(39-71) | 1.171(0.83-1.511) | 62(43-81) | 1.054(0.734-1.372) | -0.55 (-0.79 to -0.31) |
| Romania | 248(177-319) | 0.874(0.622-1.131) | 404(267-535) | 1.239(0.824-1.638) | 1.1 (0.81 to 1.39) |
| Russian Federation | 1872(1412-2269) | 1.011(0.759-1.224) | 2207(1618-2758) | 0.949(0.697-1.188) | -0.87 (-1.21 to -0.53) |
| Rwanda | 28(18-39) | 1.019(0.688-1.399) | 51(32-73) | 0.861(0.553-1.23) | -1.07 (-1.26 to -0.89) |
| Saint Kitts and Nevis | 0(0-0) | 0.366(0.227-0.546) | 0(0-0) | 0.234(0.139-0.372) | -1.23 (-1.37 to -1.08) |
| Saint Lucia | 1(0-1) | 0.75(0.491-1.042) | 1(1-2) | 0.425(0.262-0.627) | -1.88 (-2.05 to -1.71) |
| Saint Vincent and the Gredines | 0(0-1) | 0.623(0.407-0.885) | 1(0-1) | 0.519(0.342-0.775) | -0.36 (-0.61 to -0.1) |
| Samoa | 0(0-1) | 0.528(0.341-0.749) | 1(0-1) | 0.459(0.282-0.665) | -0.69 (-0.78 to -0.6) |
| San Marino | 0(0-0) | 0.609(0.398-0.854) | 0(0-0) | 0.243(0.128-0.414) | -2.21 (-2.5 to -1.93) |
| Sao Tome and Principe | 0(0-0) | 0.008(0.005-0.014) | 0(0-0) | 0.01(0.006-0.017) | 0.52 (0.45 to 0.59) |
| Saudi Arabia | 14(8-22) | 0.234(0.137-0.359) | 47(29-68) | 0.206(0.127-0.299) | -0.65 (-0.75 to -0.55) |
| Senegal | 4(2-6) | 0.121(0.081-0.177) | 11(6-16) | 0.143(0.089-0.219) | 0.28 (0.17 to 0.39) |
| Serbia | 115(76-160) | 0.975(0.638-1.356) | 127(85-179) | 0.834(0.56-1.164) | -0.52 (-0.71 to -0.33) |
| Seychelles | 1(1-2) | 2.469(1.781-3.34) | 3(2-4) | 2.663(1.943-3.484) | 0.32 (0.04 to 0.59) |
| Sierra Leone | 3(2-4) | 0.138(0.089-0.199) | 6(4-9) | 0.162(0.104-0.249) | 0.6 (0.47 to 0.73) |
| Singapore | 8(6-11) | 0.378(0.256-0.504) | 15(10-20) | 0.166(0.111-0.232) | -2.46 (-2.73 to -2.19) |
| Slovakia | 120(85-157) | 2.083(1.477-2.711) | 112(69-157) | 1.228(0.758-1.723) | -1.73 (-1.8 to -1.66) |
| Slovenia | 23(16-31) | 0.94(0.642-1.242) | 18(11-25) | 0.455(0.294-0.625) | -2.29 (-2.41 to -2.17) |
| Solomon Islands | 1(0-1) | 0.41(0.201-0.609) | 2(1-3) | 0.493(0.304-0.732) | 0.88 (0.69 to 1.08) |
| Somalia | 10(6-16) | 0.407(0.248-0.644) | 18(11-29) | 0.309(0.187-0.48) | -0.99 (-1.06 to -0.93) |
| South Africa | 231(141-322) | 1.122(0.693-1.562) | 300(218-389) | 0.648(0.468-0.831) | -2.08 (-2.26 to -1.89) |
| South Sudan | 11(7-17) | 0.448(0.283-0.681) | 13(8-20) | 0.338(0.213-0.515) | -1.14 (-1.27 to -1.01) |
| Spain | 594(434-750) | 1.155(0.847-1.459) | 408(272-555) | 0.46(0.311-0.625) | -3.06 (-3.18 to -2.95) |
| Sri Lanka | 215(169-272) | 2.324(1.827-2.899) | 483(286-721) | 1.847(1.119-2.722) | -0.37 (-0.59 to -0.16) |
| Sudan | 13(8-22) | 0.148(0.088-0.236) | 22(13-36) | 0.116(0.069-0.187) | -0.97 (-1.03 to -0.91) |
| Surime | 1(1-1) | 0.403(0.262-0.571) | 2(1-3) | 0.272(0.16-0.434) | -1.03 (-1.19 to -0.88) |
| Sweden | 66(46-89) | 0.461(0.323-0.608) | 71(47-100) | 0.336(0.228-0.467) | -0.47 (-0.73 to -0.22) |
| Switzerland | 60(42-78) | 0.612(0.435-0.798) | 68(46-97) | 0.381(0.257-0.538) | -1.63 (-1.91 to -1.34) |
| Syrian Arab Republic | 8(5-12) | 0.163(0.104-0.237) | 15(9-21) | 0.124(0.079-0.173) | -1.08 (-1.2 to -0.96) |
| Taiwan | 186(137-231) | 1.132(0.829-1.406) | 916(659-1179) | 2.231(1.607-2.857) | 2.17 (1.55 to 2.79) |
| Tajikistan | 8(5-12) | 0.309(0.174-0.457) | 8(5-14) | 0.14(0.083-0.219) | -2.73 (-2.93 to -2.53) |
| Thailand | 594(445-742) | 1.888(1.449-2.347) | 1308(926-1761) | 1.196(0.848-1.605) | -1.8 (-1.93 to -1.66) |
| Timor-Leste | 2(1-2) | 0.646(0.419-0.932) | 5(3-7) | 0.623(0.402-0.895) | -0.13 (-0.24 to -0.02) |
| Togo | 3(2-4) | 0.207(0.131-0.302) | 9(6-13) | 0.235(0.153-0.348) | 0.22 (0.13 to 0.32) |
| Tokelau | 0(0-0) | 0.397(0.243-0.601) | 0(0-0) | 0.387(0.229-0.58) | -0.07 (-0.12 to -0.03) |
| Tonga | 0(0-0) | 0.472(0.269-0.695) | 0(0-1) | 0.511(0.32-0.749) | 0.31 (0.25 to 0.37) |
| Trinidad and Tobago | 4(3-5) | 0.459(0.313-0.633) | 5(3-8) | 0.25(0.151-0.381) | -1.95 (-2.11 to -1.79) |
| Tunisia | 30(21-41) | 0.636(0.434-0.85) | 67(42-98) | 0.511(0.328-0.753) | -0.9 (-1 to -0.8) |
| Turkey | 122(81-171) | 0.35(0.231-0.492) | 191(125-268) | 0.205(0.133-0.288) | -2.25 (-2.47 to -2.04) |
| Turkmenistan | 17(12-22) | 0.853(0.617-1.115) | 21(13-31) | 0.478(0.298-0.72) | -2.2 (-2.47 to -1.93) |
| Tuvalu | 0(0-0) | 0.371(0.235-0.558) | 0(0-0) | 0.426(0.281-0.616) | 0.56 (0.52 to 0.6) |
| Uganda | 26(18-36) | 0.419(0.287-0.592) | 60(38-85) | 0.424(0.276-0.591) | -0.58 (-0.88 to -0.27) |
| Ukraine | 671(488-874) | 0.924(0.674-1.207) | 721(422-1081) | 0.991(0.573-1.493) | -0.03 (-0.42 to 0.36) |
| United Arab Emirates | 2(1-4) | 0.449(0.261-0.769) | 11(7-16) | 0.319(0.195-0.472) | 0.18 (-0.24 to 0.61) |
| United Kingdom | 567(398-727) | 0.653(0.461-0.834) | 567(369-792) | 0.452(0.298-0.62) | -1.08 (-1.23 to -0.92) |
| United Republic of Tanzania | 64(39-90) | 0.605(0.378-0.833) | 95(57-139) | 0.381(0.231-0.559) | -1.89 (-2.05 to -1.74) |
| United States Virgin Islands | 0(0-0) | 0.352(0.199-0.551) | 0(0-0) | 0.154(0.081-0.261) | -2.86 (-3.2 to -2.52) |
| United States of America | 2483(1794-3165) | 0.814(0.589-1.031) | 2427(1696-3252) | 0.418(0.293-0.557) | -2.13 (-2.28 to -1.98) |
| Uruguay | 34(23-44) | 0.879(0.593-1.139) | 32(21-44) | 0.638(0.432-0.862) | -1.15 (-1.29 to -1.01) |
| Uzbekistan | 26(17-36) | 0.215(0.142-0.305) | 94(61-130) | 0.331(0.213-0.464) | 1.26 (0.99 to 1.53) |
| Vanuatu | 0(0-0) | 0.271(0.166-0.405) | 0(0-1) | 0.249(0.162-0.361) | -0.27 (-0.32 to -0.21) |
| Venezuela (Bolivarian Republic of) | 35(26-46) | 0.385(0.279-0.505) | 74(48-109) | 0.25(0.161-0.368) | -1.7 (-1.9 to -1.5) |
| Viet m | 480(338-632) | 1.236(0.883-1.624) | 1172(824-1578) | 1.193(0.852-1.578) | -0.5 (-0.67 to -0.32) |
| Yemen | 17(10-25) | 0.344(0.21-0.505) | 38(24-54) | 0.278(0.18-0.395) | -0.88 (-0.97 to -0.79) |
| Zambia | 11(8-15) | 0.426(0.304-0.567) | 39(17-64) | 0.572(0.265-0.915) | 1.19 (0.93 to 1.45) |
| Zimbabwe | 21(14-30) | 0.55(0.366-0.784) | 43(30-59) | 0.667(0.465-0.909) | 0.87 (0.58 to 1.15) |
| **DALYs** |  |  |  |  |  |
| Afghanistan | 179(99-402) | 2.439(1.368-5.364) | 392(216-704) | 2.92(1.683-5.03) | 0.63 (0.52 to 0.74) |
| Albania | 386(249-561) | 17.315(11.289-25.203) | 649(395-993) | 15.738(9.575-24.081) | 0.09 (-0.06 to 0.24) |
| Algeria | 1117(724-1549) | 8.974(5.915-12.361) | 2262(1361-3332) | 6.099(3.797-8.972) | -1.29 (-1.4 to -1.18) |
| American Samoa | 1(1-1) | 2.98(1.962-4.252) | 2(1-3) | 3.716(2.519-5.148) | 1.54 (1.06 to 2.01) |
| Andorra | 9(6-14) | 15.564(9.403-23.852) | 12(6-19) | 7.708(4.194-12.223) | -2.08 (-2.23 to -1.92) |
| Angola | 373(218-609) | 8.29(5.016-13.477) | 1254(778-1855) | 8.954(5.497-13.05) | 0.34 (0.16 to 0.53) |
| Antigua and Barbuda | 5(3-7) | 9.919(6.215-14.229) | 8(5-11) | 6.878(4.432-9.959) | -1.13 (-1.45 to -0.8) |
| Argenti | 5785(4021-7591) | 17.703(12.302-23.224) | 5271(3572-7131) | 9.902(6.719-13.36) | -1.6 (-1.94 to -1.25) |
| Armenia | 372(275-486) | 12.242(9.077-16.006) | 550(406-703) | 12.68(9.333-16.212) | 0.39 (0.06 to 0.72) |
| Australia | 3187(2178-4174) | 16.744(11.436-21.854) | 2820(1853-4082) | 7.041(4.637-10.127) | -2.76 (-3.03 to -2.49) |
| Austria | 2553(1794-3319) | 25.48(17.977-33.024) | 2437(1641-3215) | 15.792(10.69-20.677) | -1.58 (-1.74 to -1.42) |
| Azerbaijan | 352(211-553) | 6.356(3.807-9.924) | 710(383-1159) | 6.004(3.27-9.679) | 0.14 (-0.06 to 0.34) |
| Bahamas | 26(16-37) | 15.707(9.676-22.55) | 53(32-78) | 11.748(6.997-17.225) | -0.65 (-0.87 to -0.43) |
| Bahrain | 27(18-39) | 13.895(9.507-19.486) | 95(50-147) | 8.982(4.955-13.365) | -1.54 (-1.75 to -1.33) |
| Bangladesh | 55734(37870-74611) | 109.928(74.56-146.42) | 108685(67687-158788) | 75.326(47.676-109.619) | -1.23 (-1.3 to -1.16) |
| Barbados | 29(19-42) | 10.795(6.944-15.432) | 31(18-47) | 6.095(3.566-9.209) | -1.87 (-2.03 to -1.71) |
| Belarus | 7277(5268-9272) | 55.815(40.526-70.844) | 6630(4493-9107) | 44.186(29.916-60.385) | -1.01 (-1.25 to -0.77) |
| Belgium | 3414(2440-4363) | 25.166(18.062-32.06) | 3025(2000-4072) | 15.606(10.396-20.899) | -1.8 (-2.05 to -1.54) |
| Belize | 6(4-7) | 5.948(4.162-8.029) | 15(10-21) | 4.757(3.19-6.731) | -0.35 (-0.74 to 0.05) |
| Benin | 70(48-100) | 3.474(2.378-5.056) | 188(120-291) | 3.584(2.279-5.517) | -0.15 (-0.35 to 0.04) |
| Bermuda | 13(8-19) | 20.218(13.093-29.178) | 12(8-19) | 9.857(6.182-14.631) | -1.84 (-2.16 to -1.51) |
| Bhutan | 194(121-273) | 70.178(45.009-97.819) | 356(233-517) | 56.009(37.244-80.207) | -0.86 (-0.97 to -0.74) |
| Bolivia (Pluritiol State of) | 151(94-248) | 4.451(2.759-7.421) | 305(188-485) | 3.187(1.977-5.103) | -0.73 (-0.94 to -0.51) |
| Bosnia and Herzegovi | 938(672-1235) | 19.969(14.304-26.348) | 1239(832-1718) | 21.583(14.447-29.83) | 0.66 (0.43 to 0.88) |
| Botswa | 188(126-277) | 30.592(20.68-44.481) | 418(255-617) | 25.222(15.926-35.955) | -0.77 (-0.96 to -0.58) |
| Brazil | 27938(20285-35360) | 28.918(20.967-36.798) | 35321(23880-47224) | 13.625(9.218-18.227) | -2.73 (-2.91 to -2.54) |
| Brunei Darussalam | 37(25-51) | 30.66(21.424-42.264) | 66(45-90) | 16.087(10.782-21.917) | -1.76 (-2.08 to -1.45) |
| Bulgaria | 2780(2038-3521) | 23.353(17.09-29.523) | 3359(2253-4599) | 29.815(20.137-40.675) | 0.13 (-0.2 to 0.47) |
| Burki Faso | 218(140-316) | 4.629(3.035-6.789) | 564(356-856) | 5.523(3.496-8.327) | 0.44 (0.29 to 0.58) |
| Burundi | 517(341-719) | 20.563(13.612-28.383) | 620(370-949) | 10.645(6.474-16.264) | -2.34 (-2.42 to -2.25) |
| Cabo Verde | 2(1-2) | 0.713(0.51-0.997) | 64(42-94) | 13.234(8.695-19.579) | 7.5 (4.94 to 10.13) |
| Cambodia | 1626(1139-2288) | 35.133(25.057-48.474) | 4626(3141-6341) | 36.461(25.182-49.194) | 0.03 (-0.02 to 0.08) |
| Cameroon | 224(158-319) | 4.758(3.364-6.838) | 724(458-1073) | 5.556(3.564-8.167) | 0.38 (0.2 to 0.56) |
| Cada | 8147(5918-10626) | 25.874(18.708-33.728) | 6236(4209-8544) | 9.389(6.396-12.774) | -3.15 (-3.32 to -2.99) |
| Central African Republic | 93(56-167) | 7.175(4.451-12.511) | 147(88-248) | 5.686(3.555-9.174) | -0.93 (-1.05 to -0.8) |
| Chad | 91(61-130) | 3.168(2.118-4.576) | 283(176-418) | 4.498(2.844-6.576) | 0.99 (0.77 to 1.22) |
| Chile | 843(567-1125) | 7.928(5.319-10.594) | 891(598-1212) | 3.583(2.417-4.851) | -2.48 (-2.62 to -2.35) |
| Chi | 110118(80964-140947) | 12.254(8.987-15.692) | 266689(180115-370565) | 12.17(8.277-16.854) | 0.24 (0.01 to 0.48) |
| Colombia | 1416(984-1942) | 7.747(5.338-10.639) | 1442(970-2056) | 2.593(1.743-3.7) | -4.17 (-4.4 to -3.93) |
| Comoros | 61(38-84) | 27.715(17.698-37.781) | 117(76-172) | 21.873(14.296-32.113) | -1.13 (-1.27 to -1) |
| Congo | 87(51-145) | 7.584(4.611-12.384) | 245(158-352) | 8.027(5.351-11.544) | 0.23 (0.09 to 0.37) |
| Cook Islands | 1(1-2) | 9.095(5.787-12.871) | 2(1-3) | 8.052(4.986-12.18) | -0.21 (-0.29 to -0.13) |
| Costa Rica | 172(116-233) | 9.765(6.604-13.221) | 244(155-339) | 4.393(2.779-6.113) | -2.92 (-3.08 to -2.75) |
| Croatia | 3477(2529-4448) | 53.786(38.956-68.994) | 2054(1414-2796) | 27.169(18.786-36.904) | -2.03 (-2.13 to -1.93) |
| Cuba | 2761(2004-3536) | 27.153(19.692-34.782) | 4585(3140-6151) | 23.629(16.232-31.671) | -0.3 (-0.41 to -0.19) |
| Cyprus | 104(67-145) | 13.326(8.672-18.621) | 174(110-243) | 8.933(5.716-12.456) | -1.09 (-1.16 to -1.02) |
| Czechia | 4534(3137-5933) | 35.075(24.297-45.731) | 4260(2919-5935) | 23.545(16.161-32.717) | -1.28 (-1.38 to -1.18) |
| C么te d'Ivoire | 305(192-463) | 6.427(4.137-9.629) | 873(476-1408) | 6.588(3.8-10.328) | -0.13 (-0.3 to 0.05) |
| Democratic People's Republic of Korea | 1874(1205-2654) | 10.071(6.507-14.168) | 3589(2161-5408) | 10.224(6.143-15.427) | 0.07 (-0.01 to 0.14) |
| Democratic Republic of the Congo | 1020(661-1461) | 5.823(3.774-8.247) | 2126(1348-3150) | 5.085(3.22-7.611) | -0.49 (-0.59 to -0.4) |
| Denmark | 1316(971-1631) | 18.657(13.799-23.052) | 1430(960-1893) | 13.654(9.226-18.035) | -1.75 (-2.31 to -1.17) |
| Djibouti | 34(19-56) | 20.537(11.966-33.447) | 122(67-195) | 16.822(9.752-26.224) | -0.69 (-0.8 to -0.57) |
| Dominica | 6(4-9) | 11.253(6.85-16.181) | 8(4-12) | 8.612(5.024-13.26) | -0.74 (-0.86 to -0.62) |
| Dominican Republic | 632(401-923) | 17.058(10.886-25.165) | 1477(869-2373) | 14.695(8.628-23.621) | -0.36 (-0.57 to -0.14) |
| Ecuador | 202(137-276) | 3.759(2.553-5.151) | 334(205-499) | 2.034(1.246-3.044) | -1.37 (-1.61 to -1.13) |
| Egypt | 747(525-976) | 2.539(1.791-3.312) | 2785(1871-3837) | 4.001(2.739-5.439) | 2.02 (1.53 to 2.51) |
| El Salvador | 99(65-141) | 3.249(2.117-4.623) | 190(121-282) | 3.152(2.006-4.672) | -0.03 (-0.15 to 0.08) |
| Equatorial Guinea | 13(8-21) | 5.938(3.656-9.605) | 39(22-59) | 6.664(3.921-10.407) | 0.35 (0.27 to 0.44) |
| Eritrea | 171(93-287) | 10.866(6.009-17.773) | 287(164-447) | 8.003(4.668-12.29) | -1.42 (-1.6 to -1.24) |
| Estonia | 517(368-668) | 25.631(18.262-33.115) | 485(331-652) | 22.377(15.38-30.009) | -1.06 (-1.4 to -0.73) |
| Eswatini | 33(21-50) | 10.715(7.068-16.176) | 54(32-87) | 9.051(5.501-14.377) | -0.54 (-0.81 to -0.27) |
| Ethiopia | 1090(640-1968) | 5.275(3.163-9.29) | 1729(1172-2423) | 3.959(2.674-5.545) | -0.94 (-1.08 to -0.81) |
| Fiji | 56(39-77) | 12.797(8.647-17.935) | 77(51-111) | 8.788(5.792-12.658) | -1.15 (-1.29 to -1.01) |
| Finland | 782(539-1044) | 12.062(8.367-16.063) | 701(457-967) | 7.215(4.739-9.85) | -1.5 (-1.58 to -1.41) |
| France | 32986(23215-42893) | 45.907(32.269-59.288) | 16308(10595-22119) | 14.625(9.569-19.695) | -3.66 (-3.86 to -3.46) |
| Gabon | 35(22-54) | 5.967(3.766-9.021) | 74(46-109) | 6.294(3.943-9.236) | 0.09 (-0.01 to 0.2) |
| Gambia | 17(11-25) | 4.514(2.903-6.431) | 43(28-65) | 4.117(2.638-6.086) | -0.61 (-0.78 to -0.44) |
| Georgia | 1225(854-1644) | 18.886(13.224-25.249) | 1411(1020-1818) | 25.429(18.369-32.657) | 1.8 (1.13 to 2.47) |
| Germany | 34171(24226-43435) | 30.563(21.549-38.899) | 22598(14665-30621) | 14.18(9.281-19.035) | -2.42 (-2.55 to -2.3) |
| Gha | 50(35-74) | 0.724(0.5-1.061) | 109(72-163) | 0.604(0.399-0.902) | -0.97 (-1.33 to -0.61) |
| Greece | 1858(1401-2276) | 12.831(9.719-15.703) | 2740(1952-3542) | 14.657(10.495-18.656) | 0.4 (0.27 to 0.52) |
| Greenland | 27(20-35) | 71.409(52.712-91.507) | 25(17-34) | 30.286(20.828-41.931) | -2.84 (-2.96 to -2.73) |
| Greda | 9(6-14) | 14.878(9.832-21.582) | 11(7-15) | 8.438(5.346-12.346) | -1.55 (-1.85 to -1.24) |
| Guam | 6(4-8) | 6.099(4.365-8.305) | 24(17-33) | 12.396(8.706-16.554) | 3.21 (2.98 to 3.44) |
| Guatemala | 132(88-184) | 3.815(2.532-5.397) | 214(141-303) | 1.935(1.268-2.745) | -2.76 (-2.99 to -2.53) |
| Guinea | 342(231-479) | 9.96(6.8-13.937) | 686(438-993) | 11.323(7.278-16.31) | 0.44 (0.34 to 0.53) |
| Guinea-Bissau | 12(7-18) | 2.722(1.677-4.215) | 35(23-54) | 4.307(2.783-6.474) | 1.59 (1.54 to 1.65) |
| Guya | 32(21-45) | 7.702(5.099-11.022) | 40(23-62) | 5.57(3.234-8.623) | -0.42 (-0.61 to -0.22) |
| Haiti | 289(166-529) | 7.929(4.572-14.568) | 366(206-604) | 4.429(2.482-7.22) | -1.95 (-2.08 to -1.82) |
| Honduras | 101(64-142) | 4.774(3.03-6.69) | 324(204-517) | 4.969(3.121-7.89) | 0.18 (0.12 to 0.25) |
| Hungary | 9867(7064-12884) | 72.817(52.229-94.527) | 7335(4939-9925) | 45.878(31.033-61.907) | -1.96 (-2.46 to -1.47) |
| Iceland | 47(33-61) | 18.091(12.823-23.215) | 51(34-69) | 9.773(6.54-13.191) | -2.2 (-2.35 to -2.05) |
| India | 432944(354909-522821) | 81.25(66.498-98.045) | 977501(780330-1181531) | 76.892(61.626-92.942) | -0.31 (-0.38 to -0.24) |
| Indonesia | 14215(10033-18368) | 14.207(10.216-18.291) | 38514(25454-52706) | 15.799(10.523-21.366) | 0.22 (0.13 to 0.32) |
| Iran (Islamic Republic of) | 600(404-814) | 2.093(1.41-2.828) | 1804(1236-2342) | 2.157(1.471-2.819) | 0.47 (0.32 to 0.62) |
| Iraq | 784(505-1126) | 9.277(6-13.281) | 1870(1115-2732) | 6.893(4.08-9.914) | -1.27 (-1.37 to -1.17) |
| Ireland | 1021(732-1314) | 26.269(18.779-33.767) | 677(448-933) | 9.108(6.04-12.541) | -3.38 (-3.55 to -3.21) |
| Israel | 422(302-550) | 9.265(6.672-12.069) | 606(400-832) | 5.432(3.588-7.418) | -1.87 (-2.07 to -1.66) |
| Italy | 22056(15706-27765) | 26.861(19.174-33.756) | 13237(8837-17796) | 11.12(7.47-14.784) | -2.87 (-3.04 to -2.7) |
| Jamaica | 140(95-189) | 8.318(5.678-11.221) | 174(105-269) | 5.65(3.375-8.687) | -1.5 (-1.98 to -1.02) |
| Japan | 18122(13836-22243) | 10.541(8.04-12.955) | 23313(16534-30984) | 8.299(5.959-10.816) | -1.27 (-1.7 to -0.84) |
| Jordan | 171(122-233) | 11.109(7.924-15.134) | 738(461-1061) | 8.452(5.322-12.03) | -0.85 (-0.92 to -0.78) |
| Kazakhstan | 3760(2643-4939) | 26.83(18.867-35.466) | 2934(2060-3887) | 14.57(10.212-19.332) | -2.4 (-2.65 to -2.16) |
| Kenya | 1178(697-1728) | 13.331(7.97-19.654) | 3524(2434-4743) | 13.584(9.46-18.342) | 0.06 (-0.03 to 0.14) |
| Kiribati | 21(14-29) | 49.864(34.087-68.806) | 48(30-69) | 56.769(35.256-78.864) | 0.46 (0.39 to 0.52) |
| Kuwait | 105(72-137) | 13.27(9.045-17.465) | 116(77-163) | 3.217(2.115-4.635) | -2.25 (-3.27 to -1.22) |
| Kyrgyzstan | 1576(1146-1981) | 50.125(36.406-62.964) | 917(665-1194) | 16.455(11.954-21.358) | -2 (-2.87 to -1.13) |
| Lao People's Democratic Republic | 559(374-880) | 26.287(18.016-40.297) | 1036(707-1428) | 22.049(15.315-30.208) | -0.54 (-0.61 to -0.46) |
| Latvia | 1199(881-1569) | 34.193(25.089-44.87) | 1027(705-1392) | 32.155(22.14-43.246) | -0.35 (-0.56 to -0.14) |
| Lebanon | 289(181-494) | 12.315(7.748-21.05) | 603(395-842) | 10.292(6.739-14.384) | -0.49 (-0.68 to -0.3) |
| Lesotho | 164(106-238) | 18.704(12.131-26.877) | 367(210-609) | 31.361(18.173-51.655) | 2.08 (1.87 to 2.29) |
| Liberia | 26(16-39) | 2.194(1.368-3.333) | 63(38-99) | 2.507(1.586-3.903) | 0.35 (0.23 to 0.47) |
| Libya | 192(107-297) | 9.2(5.195-14.256) | 526(329-779) | 8.349(5.262-12.4) | -0.18 (-0.25 to -0.11) |
| Lithuania | 1179(807-1525) | 26.394(18.136-34.066) | 1507(1046-2039) | 32.108(22.298-43.212) | 0.08 (-0.17 to 0.32) |
| Luxembourg | 162(112-214) | 31.888(21.951-42.179) | 124(78-170) | 12.379(7.821-17.032) | -3.06 (-3.19 to -2.94) |
| Madagascar | 1521(1048-2054) | 26.065(17.992-35.023) | 2506(1604-3662) | 16.364(10.44-23.93) | -1.6 (-1.68 to -1.53) |
| Malawi | 388(269-538) | 9.489(6.686-13.052) | 825(527-1155) | 10.246(6.713-14.013) | 0.11 (-0.01 to 0.24) |
| Malaysia | 2513(1858-3238) | 25.84(19.035-33.291) | 5905(4388-7851) | 20.138(15.015-26.542) | -1.13 (-1.35 to -0.9) |
| Maldives | 28(19-45) | 32.561(22.816-48.68) | 66(46-87) | 19.241(13.671-24.936) | -2.1 (-2.24 to -1.95) |
| Mali | 175(116-245) | 4.044(2.683-5.587) | 455(279-671) | 4.716(2.899-6.827) | 0.67 (0.56 to 0.78) |
| Malta | 74(53-98) | 17.417(12.342-23.035) | 78(51-107) | 10.641(7.129-14.481) | -1.56 (-1.71 to -1.41) |
| Marshall Islands | 2(1-3) | 8.623(5.311-14.069) | 5(3-8) | 11.016(6.182-18.169) | 1.02 (0.91 to 1.13) |
| Mauritania | 39(26-56) | 3.717(2.493-5.349) | 93(57-138) | 4.016(2.49-5.903) | 0.02 (-0.05 to 0.09) |
| Mauritius | 212(164-257) | 28.733(22.209-35.051) | 412(318-509) | 21.853(16.893-26.933) | -0.4 (-0.68 to -0.13) |
| Mexico | 2638(1882-3435) | 6.34(4.501-8.281) | 3043(2098-4098) | 2.387(1.639-3.215) | -3.38 (-3.52 to -3.23) |
| Micronesia (Federated States of) | 7(5-11) | 13.333(8.706-19.838) | 13(8-20) | 14.757(9.66-22.633) | 0.46 (0.39 to 0.52) |
| Moco | 5(3-7) | 8.611(5.416-12.307) | 5(3-8) | 6.918(4.238-10.5) | -0.7 (-0.79 to -0.6) |
| Mongolia | 206(121-305) | 18.643(10.966-27.61) | 332(199-499) | 12.242(7.303-18.536) | -1.64 (-1.91 to -1.37) |
| Montenegro | 201(142-262) | 30.15(21.324-39.495) | 269(175-375) | 28.529(18.608-39.727) | -0.22 (-0.37 to -0.07) |
| Morocco | 606(389-871) | 3.975(2.538-5.715) | 1172(691-1744) | 3.111(1.861-4.635) | -0.84 (-1 to -0.69) |
| Mozambique | 446(307-618) | 7.084(4.967-9.763) | 928(605-1296) | 7.669(5.107-10.634) | 0.58 (0.45 to 0.7) |
| Myanmar | 6797(4491-11004) | 28.117(18.867-44.343) | 9540(6571-13189) | 18.768(12.993-25.777) | -1.48 (-1.54 to -1.41) |
| mibia | 167(115-239) | 25.141(17.511-36.232) | 338(217-469) | 23.713(15.688-32.968) | -0.39 (-0.63 to -0.15) |
| uru | 1(0-1) | 13.318(7.526-23.173) | 1(0-2) | 13.484(7.144-23.747) | 0.12 (0.03 to 0.21) |
| Nepal | 8365(5725-11465) | 80.786(55.751-110.051) | 16822(11333-23621) | 68.801(46.866-95.474) | -0.52 (-0.7 to -0.33) |
| Netherlands | 3616(2668-4559) | 19.457(14.323-24.544) | 2988(2023-4047) | 9.13(6.179-12.242) | -2.3 (-2.49 to -2.1) |
| New Zealand | 579(396-770) | 15.176(10.368-20.128) | 554(372-792) | 6.949(4.69-9.816) | -2.42 (-2.75 to -2.08) |
| Nicaragua | 47(29-66) | 2.96(1.822-4.211) | 110(70-167) | 2.2(1.386-3.338) | -0.7 (-0.83 to -0.57) |
| Niger | 70(44-104) | 2.226(1.411-3.257) | 253(144-409) | 2.78(1.625-4.511) | 0.66 (0.53 to 0.79) |
| Nigeria | 488(335-700) | 1.074(0.739-1.53) | 1021(656-1533) | 1.008(0.655-1.529) | -0.21 (-0.3 to -0.12) |
| Niue | 0(0-0) | 9.318(5.768-13.639) | 0(0-0) | 9.792(6.248-14.722) | 0.05 (0.02 to 0.09) |
| North Macedonia | 443(303-581) | 21.72(14.808-28.718) | 654(429-911) | 19.454(12.804-27.122) | -0.31 (-0.54 to -0.08) |
| Northern Maria Islands | 6(4-9) | 24.325(15.665-35.655) | 27(18-38) | 44.653(29.443-61.771) | 3.02 (2.53 to 3.5) |
| Norway | 980(714-1252) | 16.501(12.104-21.015) | 603(421-804) | 6.618(4.657-8.815) | -3.33 (-3.61 to -3.06) |
| Oman | 58(33-91) | 7.231(4.158-11.47) | 113(63-177) | 4.214(2.426-6.408) | -1.33 (-1.57 to -1.1) |
| Pakistan | 96372(71186-121491) | 162.455(120.275-204.153) | 216041(160780-290779) | 156.668(118.042-208.655) | -0.38 (-0.59 to -0.18) |
| Palau | 19(13-27) | 166.871(117.345-230.093) | 38(26-52) | 147.262(102.825-201.803) | -0.36 (-0.39 to -0.32) |
| Palestine | 38(24-56) | 4.292(2.746-6.363) | 100(67-140) | 3.628(2.448-5.109) | -0.72 (-0.83 to -0.62) |
| Pama | 119(82-161) | 8.067(5.559-10.862) | 174(111-255) | 3.935(2.52-5.78) | -2.52 (-2.71 to -2.33) |
| Papua New Guinea | 177(94-267) | 7.592(4.055-11.419) | 644(365-984) | 8.999(5.115-13.709) | 0.62 (0.5 to 0.74) |
| Paraguay | 349(235-481) | 15.374(10.28-21.072) | 850(536-1283) | 14.148(8.952-21.358) | -0.47 (-0.68 to -0.26) |
| Peru | 248(165-352) | 1.998(1.307-2.879) | 525(311-813) | 1.538(0.908-2.396) | -1.17 (-1.36 to -0.98) |
| Philippines | 6896(4719-8986) | 21.864(14.84-28.364) | 13079(9408-17247) | 15.026(10.945-19.674) | -1.45 (-1.61 to -1.29) |
| Poland | 14458(11041-17772) | 33.861(25.841-41.623) | 19155(13582-24859) | 30.491(21.79-39.521) | -0.29 (-0.39 to -0.19) |
| Portugal | 3389(2290-4483) | 25.909(17.583-34.246) | 3131(2145-4229) | 17.017(11.729-22.74) | -1.23 (-1.41 to -1.05) |
| Puerto Rico | 594(376-886) | 16.597(10.494-24.762) | 380(223-599) | 6.268(3.673-9.796) | -2.84 (-3.04 to -2.63) |
| Qatar | 8(4-12) | 4.833(2.848-7.453) | 57(29-92) | 3.908(1.889-6.408) | -0.25 (-0.75 to 0.26) |
| Republic of Korea | 3819(2755-4846) | 11.357(8.184-14.563) | 5939(3813-8278) | 6.428(4.139-8.946) | -2.39 (-2.68 to -2.1) |
| Republic of Moldova | 1786(1268-2296) | 38.052(27.051-48.919) | 1886(1315-2448) | 32.935(23.106-42.758) | -0.69 (-0.93 to -0.44) |
| Romania | 7968(5746-10141) | 28.071(20.124-35.847) | 12133(8065-16039) | 39.199(26.179-51.86) | 1.05 (0.73 to 1.38) |
| Russian Federation | 60160(45426-72776) | 32.684(24.707-39.522) | 67765(50172-84428) | 30.306(22.467-37.822) | -0.96 (-1.3 to -0.61) |
| Rwanda | 801(521-1145) | 26.264(17.342-37.222) | 1408(861-2070) | 20.788(12.928-30.103) | -1.35 (-1.56 to -1.15) |
| Saint Kitts and Nevis | 3(2-5) | 9.912(6.149-14.494) | 5(3-7) | 5.916(3.552-9.085) | -1.49 (-1.64 to -1.34) |
| Saint Lucia | 17(12-24) | 20.076(13.356-27.63) | 28(17-41) | 11.311(6.923-16.475) | -1.85 (-1.99 to -1.7) |
| Saint Vincent and the Gredines | 12(8-16) | 16.803(11.132-23.486) | 21(13-30) | 13.937(8.952-20.638) | -0.37 (-0.61 to -0.13) |
| Samoa | 14(9-20) | 14.783(9.604-21.278) | 20(12-30) | 12.936(7.852-18.701) | -0.66 (-0.75 to -0.57) |
| San Marino | 6(4-8) | 17.437(11.356-24.31) | 4(2-7) | 7.087(3.709-12.225) | -2.18 (-2.45 to -1.91) |
| Sao Tome and Principe | 0(0-0) | 0.176(0.115-0.265) | 0(0-0) | 0.214(0.134-0.338) | 0.51 (0.35 to 0.66) |
| Saudi Arabia | 458(274-703) | 6.57(3.87-10.156) | 1643(1018-2403) | 5.78(3.626-8.49) | -0.63 (-0.72 to -0.53) |
| Senegal | 116(73-174) | 3.324(2.129-4.941) | 303(183-442) | 3.597(2.195-5.371) | -0.02 (-0.15 to 0.11) |
| Serbia | 3508(2337-4812) | 28.556(18.901-39.258) | 3571(2393-4981) | 24.914(16.903-34.434) | -0.46 (-0.68 to -0.25) |
| Seychelles | 35(24-47) | 62.342(43.855-85.06) | 77(55-103) | 63.089(45.86-83.284) | 0.15 (-0.11 to 0.4) |
| Sierra Leone | 77(47-110) | 3.671(2.275-5.256) | 171(110-253) | 4.187(2.723-6.241) | 0.51 (0.38 to 0.64) |
| Singapore | 242(165-320) | 10.053(6.821-13.278) | 366(249-493) | 4.103(2.782-5.546) | -2.65 (-2.9 to -2.4) |
| Slovakia | 3858(2743-5044) | 67.777(48.401-88.761) | 3265(2009-4624) | 37.358(22.882-52.485) | -2.01 (-2.1 to -1.93) |
| Slovenia | 761(523-999) | 31.04(21.328-40.701) | 517(336-705) | 14.307(9.388-19.407) | -2.45 (-2.57 to -2.32) |
| Solomon Islands | 18(8-28) | 11.349(5.405-17.248) | 61(38-93) | 14.273(8.851-21.282) | 1.05 (0.85 to 1.25) |
| Somalia | 317(183-525) | 10.961(6.581-17.724) | 575(340-898) | 8.155(4.903-12.781) | -1.12 (-1.19 to -1.04) |
| South Africa | 6862(4137-9576) | 30.993(18.738-43.295) | 8672(6190-11416) | 17.335(12.453-22.672) | -2.17 (-2.34 to -2) |
| South Sudan | 315(194-494) | 11.786(7.291-18.228) | 377(227-590) | 8.558(5.29-13.319) | -1.29 (-1.45 to -1.13) |
| Spain | 17893(13284-22426) | 36.473(27.049-45.864) | 11177(7671-15095) | 13.656(9.428-18.361) | -3.33 (-3.48 to -3.18) |
| Sri Lanka | 5348(4162-6806) | 49.889(38.984-63.416) | 11926(6876-18132) | 43.523(25.331-65.532) | 0.05 (-0.21 to 0.31) |
| Sudan | 393(238-643) | 3.922(2.357-6.343) | 662(395-1052) | 2.96(1.788-4.785) | -1.14 (-1.21 to -1.06) |
| Surime | 30(19-41) | 10.853(7.024-15.225) | 51(30-80) | 7.511(4.45-11.904) | -0.95 (-1.11 to -0.8) |
| Sweden | 1587(1128-2066) | 12.186(8.751-15.801) | 1569(1071-2160) | 8.463(5.864-11.6) | -0.64 (-0.91 to -0.38) |
| Switzerland | 1626(1170-2097) | 17.668(12.726-22.794) | 1576(1058-2209) | 9.753(6.57-13.558) | -2.09 (-2.4 to -1.77) |
| Syrian Arab Republic | 225(148-319) | 4.015(2.604-5.755) | 403(249-566) | 2.861(1.802-3.977) | -1.32 (-1.43 to -1.21) |
| Taiwan | 6175(4572-7671) | 35.216(26.175-43.686) | 27920(19981-35878) | 70.561(50.719-90.516) | 2.09 (1.42 to 2.78) |
| Tajikistan | 233(138-339) | 8.092(4.758-11.747) | 259(153-424) | 3.758(2.233-6.053) | -2.65 (-2.81 to -2.49) |
| Thailand | 15277(11263-19348) | 42.119(31.541-53.078) | 28633(19678-39372) | 26.233(18.086-35.898) | -1.89 (-2 to -1.78) |
| Timor-Leste | 42(26-62) | 14.621(9.202-21.104) | 120(78-176) | 14.049(9.032-20.558) | -0.13 (-0.26 to 0.01) |
| Togo | 75(47-112) | 5.5(3.47-8.167) | 268(172-399) | 6.07(3.913-8.931) | 0.12 (0.01 to 0.22) |
| Tokelau | 0(0-0) | 11.048(6.811-16.698) | 0(0-0) | 11.184(6.626-16.762) | 0.06 (0.01 to 0.11) |
| Tonga | 7(4-10) | 12.072(6.991-17.951) | 11(7-16) | 13.315(8.246-19.812) | 0.39 (0.34 to 0.45) |
| Trinidad and Tobago | 103(71-139) | 12.074(8.389-16.34) | 135(81-202) | 6.839(4.116-10.216) | -1.85 (-2.01 to -1.69) |
| Tunisia | 817(553-1121) | 15.571(10.628-21.13) | 1710(1059-2562) | 12.416(7.731-18.423) | -0.93 (-1.02 to -0.85) |
| Turkey | 3609(2368-5042) | 9.423(6.268-13.162) | 5169(3409-7233) | 5.31(3.49-7.45) | -2.35 (-2.54 to -2.15) |
| Turkmenistan | 541(395-700) | 25.489(18.597-33.004) | 657(415-979) | 14.01(8.81-21.011) | -2.29 (-2.56 to -2.02) |
| Tuvalu | 1(0-1) | 10.637(6.725-16.241) | 1(1-2) | 12.268(8.101-17.862) | 0.6 (0.55 to 0.64) |
| Uganda | 740(508-1048) | 10.776(7.336-14.98) | 1736(1084-2467) | 10.662(6.799-15.171) | -0.73 (-1.05 to -0.4) |
| Ukraine | 21102(15289-27770) | 29.522(21.439-39.072) | 22511(12878-33873) | 32.259(18.301-48.737) | -0.01 (-0.4 to 0.39) |
| United Arab Emirates | 78(46-131) | 11.592(6.597-20.285) | 404(237-605) | 6.728(4.105-9.799) | -0.82 (-1.19 to -0.44) |
| United Kingdom | 14181(10126-17859) | 17.576(12.624-22.032) | 13751(9168-18746) | 12.099(8.151-16.357) | -1.06 (-1.19 to -0.94) |
| United Republic of Tanzania | 1824(1105-2586) | 15.683(9.675-22.067) | 2689(1587-3979) | 9.677(5.78-14.255) | -1.97 (-2.13 to -1.82) |
| United States Virgin Islands | 9(5-14) | 9.672(5.467-14.956) | 7(4-12) | 4.134(2.191-6.991) | -2.89 (-3.2 to -2.57) |
| United States of America | 68138(49856-85428) | 23.449(17.214-29.312) | 61990(43363-81617) | 11.336(7.966-14.85) | -2.31 (-2.46 to -2.16) |
| Uruguay | 967(655-1239) | 26.009(17.645-33.246) | 875(598-1164) | 18.362(12.607-24.39) | -1.27 (-1.41 to -1.12) |
| Uzbekistan | 809(539-1131) | 6.55(4.355-9.114) | 2929(1914-4045) | 9.459(6.12-13.086) | 0.97 (0.7 to 1.24) |
| Vanuatu | 5(3-8) | 7.35(4.595-11.157) | 15(10-22) | 7.036(4.653-10.526) | -0.15 (-0.2 to -0.09) |
| Venezuela (Bolivarian Republic of) | 931(692-1203) | 9.355(6.876-12.121) | 1856(1197-2742) | 6.004(3.878-8.844) | -1.76 (-1.96 to -1.57) |
| Viet m | 12386(8536-16611) | 30.407(21.239-40.527) | 32114(21851-43567) | 30.025(20.684-40.439) | -0.34 (-0.49 to -0.2) |
| Yemen | 501(309-784) | 9.128(5.646-13.944) | 1116(717-1599) | 6.98(4.494-10.009) | -1.07 (-1.16 to -0.97) |
| Zambia | 319(224-436) | 10.608(7.573-14.255) | 1155(458-1948) | 14.861(6.33-24.546) | 1.36 (1.07 to 1.66) |
| Zimbabwe | 573(372-821) | 13.499(8.788-19.312) | 1276(851-1766) | 16.774(11.488-22.936) | 0.89 (0.6 to 1.19) |
